# Accumulation of **α**-synuclein mediates podocyte injury in Fabry nephropathy

**DOI:** 10.1172/JCI157782

**Published:** 2023-06-01

**Authors:** Fabian Braun, Ahmed Abed, Dominik Sellung, Manuel Rogg, Mathias Woidy, Oysten Eikrem, Nicola Wanner, Jessica Gambardella, Sandra D. Laufer, Fabian Haas, Milagros N. Wong, Bernhard Dumoulin, Paula Rischke, Anne Mühlig, Wiebke Sachs, Katharina von Cossel, Kristina Schulz, Nicole Muschol, Sören W. Gersting, Ania C. Muntau, Oliver Kretz, Oliver Hahn, Markus M. Rinschen, Michael Mauer, Tillmann Bork, Florian Grahammer, Wei Liang, Thorsten Eierhoff, Winfried Römer, Arne Hansen, Catherine Meyer-Schwesinger, Guido Iaccarino, Camilla Tøndel, Hans-Peter Marti, Behzad Najafian, Victor G. Puelles, Christoph Schell, Tobias B. Huber

**Affiliations:** 1 III. Department of Medicine and; 2 Hamburg Center for Kidney Health, University Medical Center Hamburg-Eppendorf, Hamburg, Germany.; 3 Department of Medicine IV and; 4 Institute of Surgical Pathology, Medical Center–University of Freiburg, Faculty of Medicine, University of Freiburg, Freiburg, Germany.; 5 University Children’s Hospital and; 6 University Children’s Research, University Medical Center Hamburg-Eppendorf, Hamburg, Germany.; 7 Department of Clinical Medicine, University of Bergen, Bergen, Norway.; 8 Department of Medicine, Haukeland University Hospital, Bergen, Norway.; 9 Department of Advanced Biomedical Sciences and; 10 Interdepartmental Center of Research on Hypertension and Related Conditions, Federico II University, Napoli, Italy.; 11 Department of Clinical Medicine, Aarhus University, Aarhus, Denmark.; 12 Department of Pathology, Aarhus University Hospital, Aarhus, Denmark.; 13 Institute of Cellular and Integrative Physiology, Center for Experimental Medicine, and; 14 Department of Pediatrics, International Center for Lysosomal Disorders (ICLD), University Medical Center Hamburg-Eppendorf, Hamburg, Germany.; 15 Department of Biomedicine and; 16 Aarhus Institute for Advanced Studies, Aarhus University, Aarhus, Denmark.; 17 Departments of Pediatrics and Medicine, University of Minnesota, Minneapolis, Minnesota, USA.; 18 Faculty of Biology and Signalling Research Centres BIOSS and CIBSS, University of Freiburg, Freiburg, Germany.; 19 Department of Vascular and Endovascular Surgery, University Hospital of Münster, Münster, Germany.; 20 Freiburg Institute for Advanced Studies, University of Freiburg, Freiburg, Germany.; 21 Department of Experimental Pharmacology and Toxicology, University Medical Center Hamburg-Eppendorf, Hamburg, Germany.; 22 German Center for Heart Research (DZHK), partner site Hamburg/Lübeck/Kiel, Germany.; 23 Department of Pediatrics, Haukeland University Hospital, Bergen, Norway.; 24 Department of Laboratory Medicine and Pathology, University of Washington, Seattle, Washington, USA.

**Keywords:** Genetics, Nephrology, Chronic kidney disease, Drug therapy, Genetic diseases

## Abstract

Current therapies for Fabry disease are based on reversing intracellular accumulation of globotriaosylceramide (Gb3) by enzyme replacement therapy (ERT) or chaperone-mediated stabilization of the defective enzyme, thereby alleviating lysosomal dysfunction. However, their effect in the reversal of end-organ damage, like kidney injury and chronic kidney disease, remains unclear. In this study, ultrastructural analysis of serial human kidney biopsies showed that long-term use of ERT reduced Gb3 accumulation in podocytes but did not reverse podocyte injury. Then, a CRISPR/Cas9–mediated α-galactosidase knockout podocyte cell line confirmed ERT-mediated reversal of Gb3 accumulation without resolution of lysosomal dysfunction. Transcriptome-based connectivity mapping and SILAC-based quantitative proteomics identified α-synuclein (SNCA) accumulation as a key event mediating podocyte injury. Genetic and pharmacological inhibition of SNCA improved lysosomal structure and function in Fabry podocytes, exceeding the benefits of ERT. Together, this work reconceptualizes Fabry-associated cell injury beyond Gb3 accumulation, and introduces SNCA modulation as a potential intervention, especially for patients with Fabry nephropathy.

## Graphical abstract

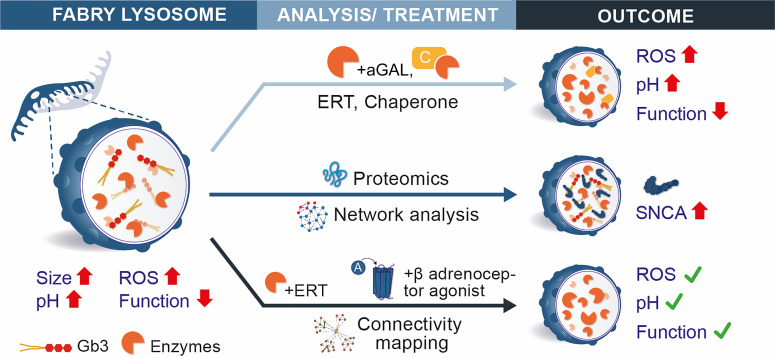

## Introduction

Anderson-Fabry disease (FD) is an X-linked lysosomal storage disorder caused by mutations in the *GLA* gene (1), which results in an impairment of the hydrolase α-galactosidase A (aGAL). This enzyme deficiency leads to lysosomal dysfunction (2) via progressive accumulation of globotriaosylceramide (Gb3) and other glycosphingolipids (3) in most cells of the body (4). Patients with FD suffer from extensive and progressive end-organ damage — for example, cardiomyopathy and nephropathy, both of which are key factors affecting long-term survival (5).

The first therapies became available in 2001 with enzyme replacement therapy (ERT) (6, 7) and were complemented by chaperone therapy in 2016 (8). All have proven efficient in decreasing Gb3 deposits (9–14), but their impact on the reversal of end-organ damage remains unclear.

Glomerular epithelial cells (podocytes) are primary targets in chronic kidney diseases that progress to the requirement of dialysis or transplantation (15–18). Since podocytes exhibit limited regenerative capacities (19, 20), injury and loss are considered critical steps in renal pathophysiology and central therapeutic targets. Previous studies have shown podocytes to accumulate the highest amount of Gb3 in Fabry nephropathy, resulting in early (micro)albuminuria (21, 22). However, the molecular mechanisms of Fabry podocytopathy remain elusive, in part because of limited access to human biopsy material. Furthermore, existing animal models do not completely reflect the human phenotype, especially those observed in Fabry nephropathy (23–25).

In this study, we report prevailing signs of podocyte damage in human kidney biopsies before and after ERT despite significant Gb3 reduction. We established a CRISPR/Cas9–based *GLA* knockout (KO) in human podocytes, which recapitulates classical cell injury features like lysosomal dysfunction. Quantitative proteomics combined with a network medicine approach identified the accumulation of α-synuclein (SNCA) as a mediator of lysosomal dysfunction resistant to ERT both in vitro and in patient biopsies. To our knowledge, this is the first report showing that accumulation of SNCA directly contributes to lysosomal impairment and disease severity in FD in a substrate-independent fashion, which suggests that pharmacological targeting of SNCA could serve as an additional therapeutic strategy, especially for patients with Fabry nephropathy.

## Results

### Podocyte injury persists despite ERT and significant reductions in Gb3 deposits.

**Figure 1:**
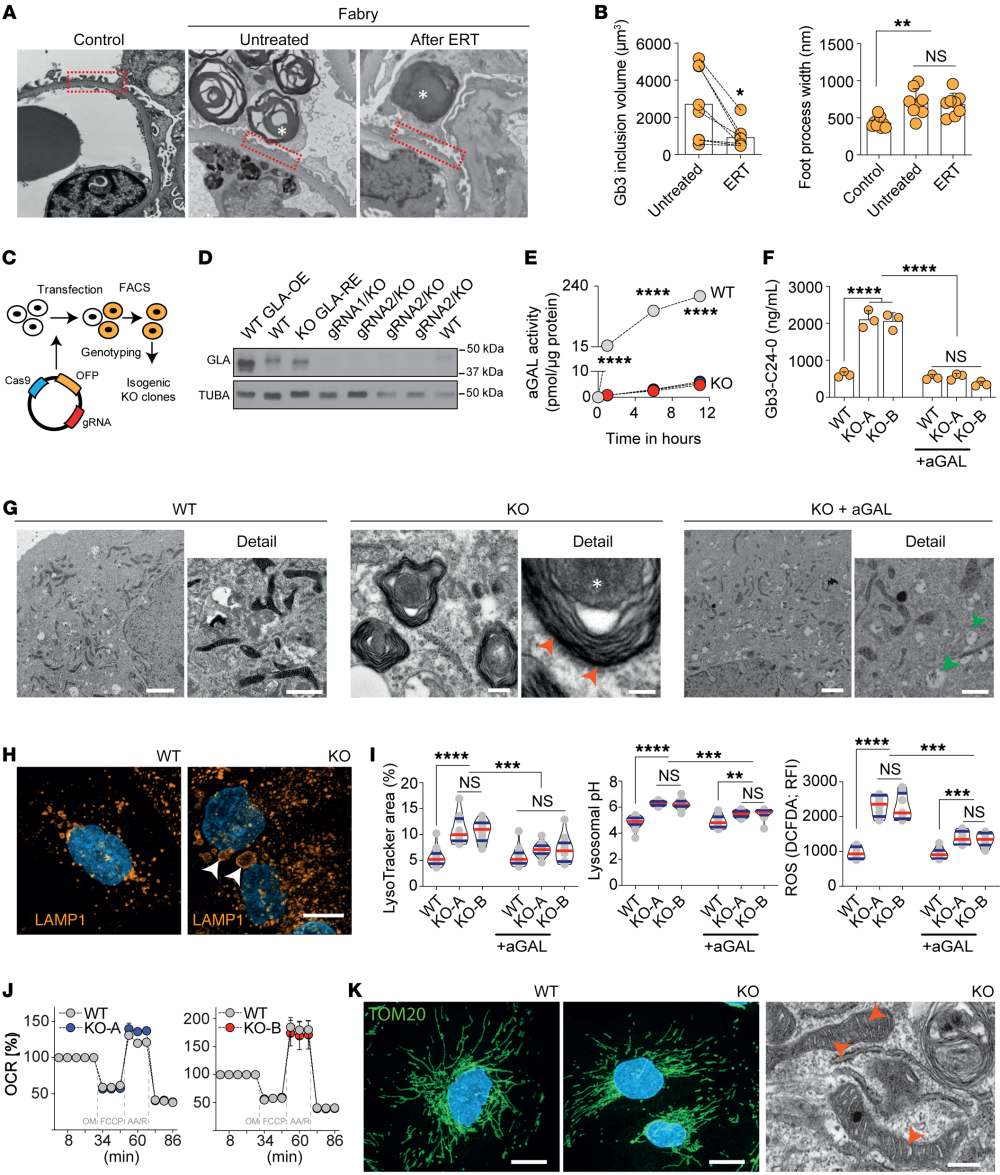
Podocytes in Fabry disease show persistent lysosomal dysfunction and damage despite enzyme replacement therapy. (**A**) Transmission electron microscopy (TEM) comparison of foot processes between control and Fabry kidney biopsy before and after ERT with many foot processes widened in Fabry biopsies both untreated and after ERT. Asterisks show Gb3 inclusions in podocytes. Original magnification, ×52,800. (**B**) Significant decrease of podocyte Gb3 inclusions after ERT but persistence of increased foot process width. (**C**) Schematic overview of *GLA*-knockout (KO) podocyte generation by CRISPR/Cas9 genome editing. (**D**) Western blots show a complete absence of GLA expression in several *GLA*-KO clones. (**E**) Abolished GLA activity in 2 KO clones compared with WT cells. (**F**) Mass spectrometry analysis confirms the accumulation of Gb3-C24-0 isoform in KO cells, normalized upon 96 hours of α-galactosidase therapy (*n* = 3). (**G**) TEM shows zebra bodies exclusively in *GLA*-KO clones (red arrowheads). While WT cells depict a normal ultrastructure, aGAL-treated KO cells have remnant vacuoles (green arrowheads) without zebra bodies. Scale bars: 1 μm. (**H**) Lysosomal visualization using LAMP1 staining in differentiated WT and KO cells reveals an increased number and size (arrowheads) of lysosomes in the *GLA*-KO cells. Scale bar: 10 μm. (**I**) Quantification of lysosomal area (*n* = 14), pH (*n* = 12), and ROS production (*n* = 8). (**J**) Seahorse XFp experiments confirm normal mitochondrial function in KO cells (*n* = 8). OCR, oxygen consumption rate. (**K**) Mitochondrial import receptor subunit (TOM20) staining in WT and KO cells is equally abundant and normally distributed. TEM images confirm a normal mitochondrial ultrastructure in KO cells. Scale bars: 10 μm in immunofluorescence, 500 nm in electron microscopy. Violin plots indicate median (red) and upper and lower quartile (blue). *P < 0.05, **P < 0.01, ***P < 0.001, ****P < 0.0001. One-way ANOVA with Tukey’s multiple-comparison test.

Ultrastructural analysis of serial human kidney biopsies (see Supplemental Table 1 for full demographics; supplemental material available online with this article; https://doi.org/10.1172/JCI157782DS1) revealed typical accumulation of Gb3 within podocytes combined with classical signs of podocyte injury, including alterations in foot process morphology (Figure 1A). ERT partially reversed this phenotype by reducing podocyte-specific Gb3 inclusions. Importantly, increased foot process width remained unaffected (Figure 1B), highlighting the possibility of alternative pathomechanisms in Fabry podocytopathy.

### Generation of a GLA-deficient podocyte line using CRISPR/Cas9 genome editing.

Next, we developed an in vitro system to model FD-related podocyte damage. Using 2 independent guide RNAs (gRNAs), the first exon within the *GLA* locus was targeted to generate *GLA*-deficient immortalized male human podocytes (26) with CRISPR/Cas9 (Figure 1C) (27, 28). Subclones with mutations resulting in premature stop codons (Supplemental Figure 1A) and efficient knockout on the protein level (Figure 1C) were selected for further analyses. aGAL protein levels and activity were almost completely abolished (Figure 1, D and E), resulting in a significant increase of Gb3 in both thin-layer chromatography (Supplemental Figure 1B) and lipid mass spectrometry (Figure 1F and Supplemental Figure 1, C–E) that decreased to baseline after treatment with recombinant aGAL. Multilaminar inclusions (zebra bodies) were detected within *GLA*-deficient podocytes with almost complete clearance of these structures after aGAL treatment (Figure 1G).

### In vitro ERT does not fully revert podocyte injury.

Both lysosome number and size were dramatically increased in *GLA*-KO clones, and the increase was partially reversed by aGAL treatment (Figure 1, H and I). These structural abnormalities were associated with impairment of lysosomal function. Recombinant aGAL treatment did not modify lysosomal pH or oxidative stress via reactive oxygen species (ROS). As the latter could be a result of mitochondrial dysfunction, we investigated oxygen consumption rate (Figure 1J) and mitochondrial morphology (Figure 1K) and detected no differences between WT and KO clones.

Podocytes are known for their prominent autophagy features (29), and we detected an increase in autophagy via the decrease of surrogate marker p62, while LC3-II was unchanged both at baseline and in chloroquine-challenged KO cells (Supplemental Figure 2). Together, our data suggest that podocyte injury extends beyond substrate accumulation, as multiple features of FD are only ameliorated by aGAL replacement.

### SNCA accumulates in GLA-deficient podocytes and is resistant to short-term ERT.

**Figure 2:**
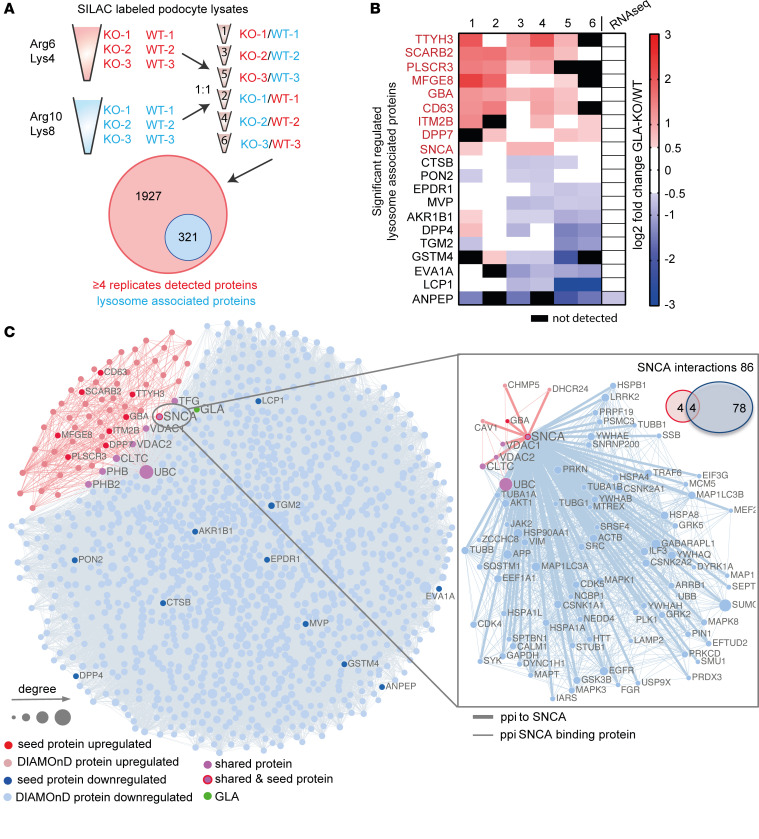
SILAC-based proteomics and network analyses identify SNCA accumulation as a potential pathogenic pathway. (**A**) Schematic overview of mass spectrometry analysis using SILAC-labeled WT and KO clones. Mass spectrometry yielded 2,248 proteins, among which 321 are lysosome-enriched. (**B**) The top 10 up- and downregulated lysosome-enriched proteins. (**C**) Network-based analysis of up- and downregulated lysosomal proteins associated with *GLA* knockout. Nodes represent genes and are connected if there is a known protein interaction between them. The node size is proportional to the number of its connections. Red and blue nodes represent up- and downregulated seed proteins, respectively. Light red and light blue nodes represent the respective DIAMOnD proteins. GLA is depicted as a green node. Pink nodes indicate shared proteins between the 2 modules. The separate network and Venn diagram on the right show the number and interaction partners of SNCA.

We used SILAC-based quantitative proteomics and transcriptome profiling via RNA sequencing to determine potential alterations in gene expression and protein abundance underlying the observed lysosomal dysfunction (Supplemental Figure 3A). Filtering for lysosome-associated proteins resulted in the detection of 321 differentially expressed proteins (Figure 2A). Surprisingly, the top 20 regulated lysosomal proteins (except for ANPEP) were not altered at a transcriptomic level (Figure 2B).

Next, we used a network medicine approach to further evaluate how these proteins are functionally related to each other. We identified the 2 podocyte-specific *GLA*-KO modules for the respective up- and downregulated lysosomal proteins (Figure 2B and Supplemental Figure 3, B and C). The upregulated hits resulted in a smaller, more specific module (64 proteins; *z* score: 28). Gene Ontology (GO) enrichment for cellular compartment and reactome pathways revealed an involvement of processes associated with membrane trafficking, autophagy, mitophagy, and the lysosome in both modules (Supplemental Figure 4, A and B). Molecular function GO terms overrepresented in the disease modules were associated with proteins binding to phosphorylated residues and β-adrenergic signaling kinases (Supplemental Figure 4C).

Strikingly, α-synuclein (SNCA) was the only protein found as an upregulated protein by the SILAC-based proteome analysis but also found by the network-based approach as a protein residing in the downregulated module, connecting both the upregulated and downregulated disease modules as a seed protein. SNCA binds to 4 of the 8 proteins that connect both modules with each other, and the known interaction partners of SNCA are involved in autophagosome and lysosome function, such as GABARAPL1, MAPK1, LAMP2, and SQSTM1 (p62) (Figure 2C).

**Figure 3:**
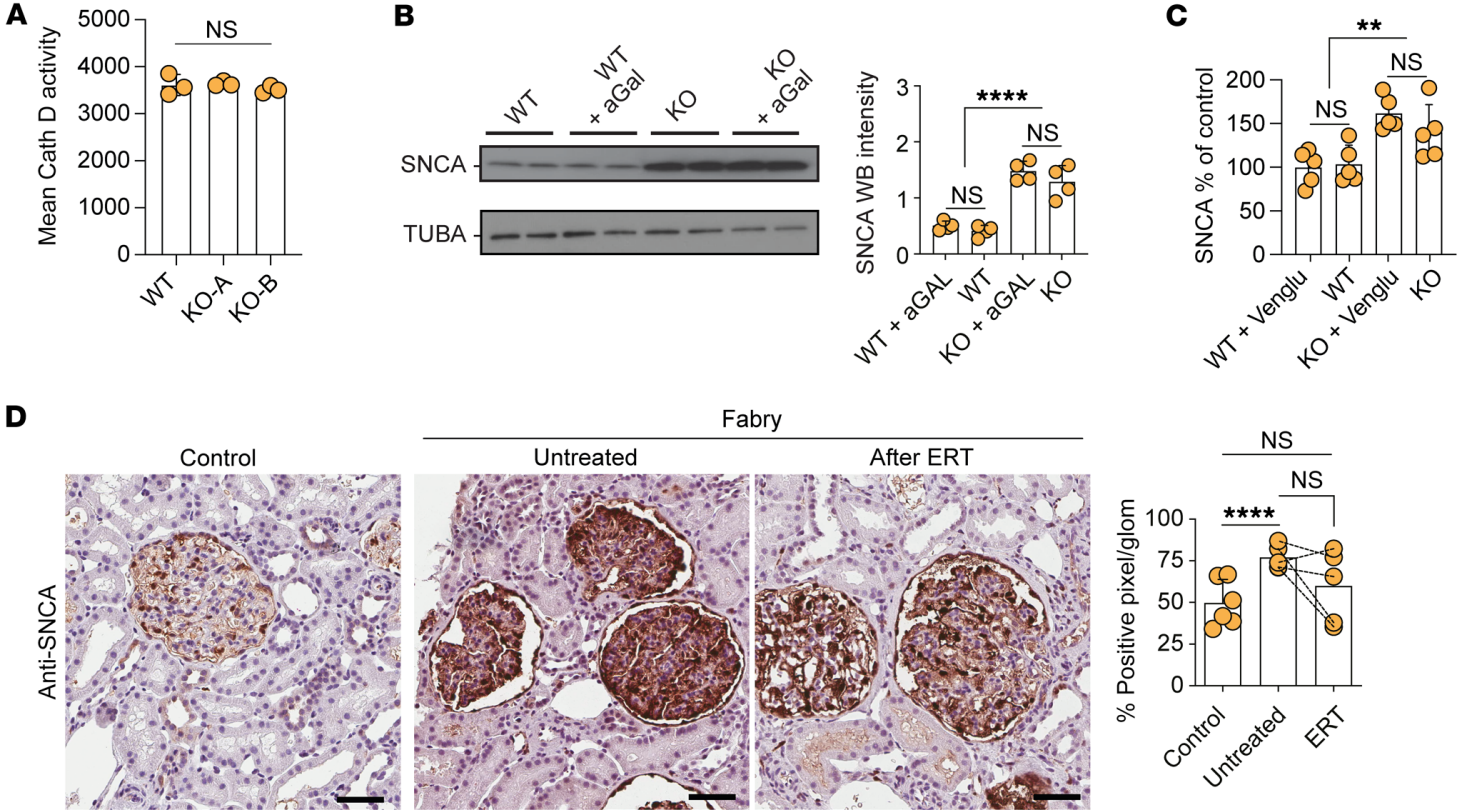
SNCA accumulation is resistant to enzyme replacement and substrate reduction therapy. (**A**) Mean cathepsin D activity in WT cells and 2 KO clones showing no differences in enzyme activity (*n* = 3). (**B**) Western blots of SNCA and TUBA in vehicle- and aGAL-treated WT and KO cells with quantification confirming the overexpression of SNCA protein and its resistance to aGAL treatment (*n* = 4). (**C**) Human SNCA ELISA depicting SNCA accumulation in KO clones and resistance to substrate reduction therapy using Venglustat (*n* = 5). (**D**) SNCA staining in representative images and quantification of human renal biopsies showing increase in untreated Fabry samples with resistant accumulation in patients who underwent 5 years of ERT (*n* = 5). Scale bars: 50 μm. **P < 0.01, ****P < 0.0001. One-way ANOVA with Tukey’s multiple-comparison test.

One major degradation pathway for SNCA depends on the enzyme cathepsin D (30, 31), yet Fabry podocytes did not show altered cathepsin D activity (Figure 3A). Treatment with recombinant aGAL for 96 hours mitigated and normalized the expression levels of all top 10 upregulated lysosomal proteins except for SNCA (Figure 3B and Supplemental Figure 5). Similarly, substrate reduction therapy using an inhibitor of glucosylceramide synthase did not result in a significant decrease of SNCA protein levels in our cell culture model, as well as in several organs of Fabry mice (Figure 3C and Supplemental Figure 6, A and B). In addition, chaperone treatment also did not change organ-specific SNCA levels in vivo.

In human renal biopsies, SNCA was detected almost exclusively in the glomerular compartment, showing an increased expression in samples of untreated Fabry patients (Figure 3D). SNCA staining intensity in biopsies of patients who underwent 5 years of ERT was partially reduced compared with baseline levels (Figure 3D). Remarkably, we did not observe different SNCA levels in patient-derived primary urinary cells, and no induction was seen through challenging of these cells with globotriaosyl-sphingosine (lyso-Gb3), the main degradation product of Gb3, implicated in the disease’s molecular pathology (Supplemental Figure 7, A and B).

### Modulating SNCA accumulation ameliorates Fabry podocytopathy.

**Figure 4:**
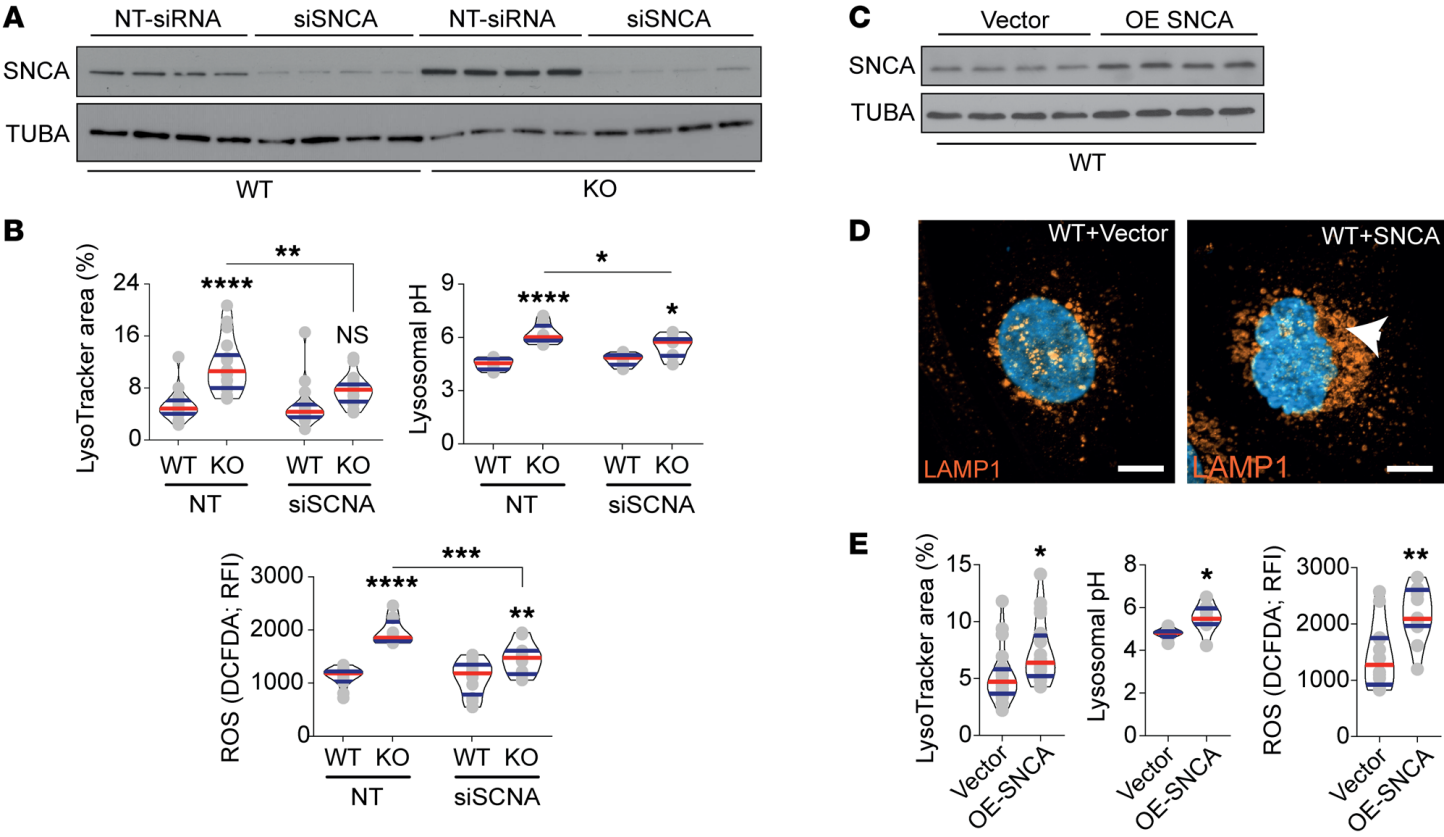
SNCA mediates observed lysosomal dysfunction. (**A**) Representative Western blot confirming the efficacy of siRNA targeting SNCA in WT and KO clones (*n* = 4). (**B**) Quantification of lysosomal area, pH, and ROS production upon SNCA siRNA treatment (*n* = 18). (**C**) Representative Western blot confirming the overexpression of SNCA in WT cells (*n* = 4). (**D**) LAMP1 immunofluorescence staining shows an increase in lysosomal aggregation upon SNCA overexpression (arrowhead). Scale bars: 10 μm. (**E**) Quantification of lysosomal area (*n* = 20), pH (*n* = 8), and ROS production (*n* = 12) upon SNCA overexpression. Violin plots indicate median (red) and upper and lower quartile (blue). *P < 0.05, **P < 0.01, ***P < 0.001, ****P <0.0001. One-way ANOVA with Tukey’s multiple-comparison test (**B**); unpaired, 2-tailed Student’s *t* test (**E**).

To elucidate the functional effect of alterations in SNCA levels in WT and aGAL-deficient cells, we performed knockdown and overexpression analyses. We achieved a strong inhibition in both KO and WT cells 48 hours after siRNA transfection (Figure 4A). This inhibition was associated with a significant reduction in lysosomal area, lysosomal pH, and ROS accumulation (Figure 4B) without complete reversal to WT levels. Next, a reverse overexpression of SNCA in WT cells (Figure 4C) induced pronounced alterations of lysosomal structure (Figure 4D), and marked increases in lysosomal area, pH, and ROS production (Figure 4E), mirroring the Fabry phenotype and confirming a central role of SNCA signaling as a Gb3-independent mechanism of podocyte injury.

### β_2_-Adrenergic receptor agonists as therapy for Fabry podocytopathy.

**Figure 5:**
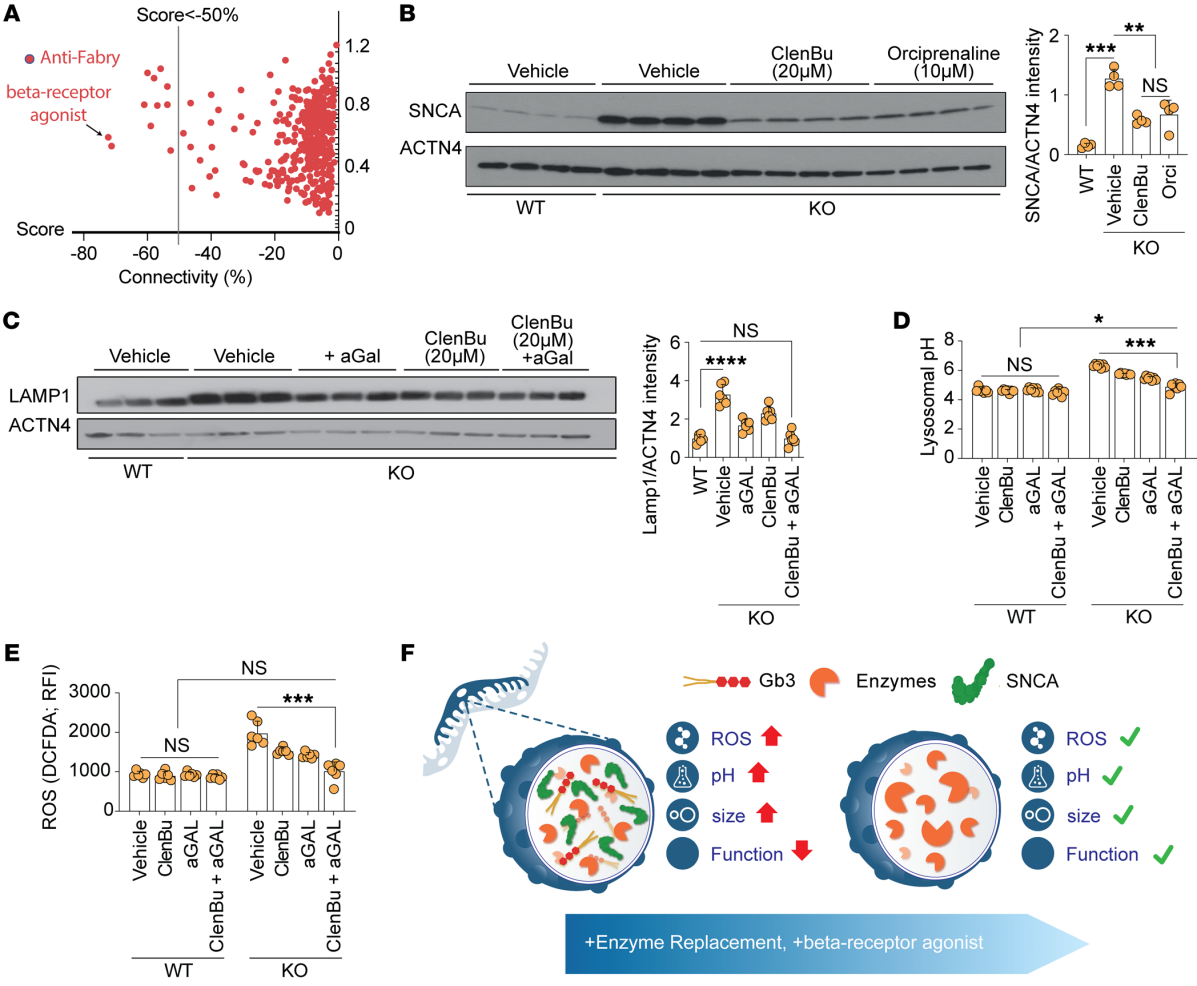
β_2_-Adrenergic receptor agonists decrease SNCA protein levels and ameliorate lysosomal dysfunction. (**A**) Connectivity mapping showing anti-Fabry compounds, with the β_2_-adrenergic receptor agonist orciprenaline exhibiting the highest score. (**B**) Western blots show the expression of SNCA in WT and untreated *GLA*-KO cells and KO cells treated with 20 μM clenbuterol and 10 μM orciprenaline (*n* = 6). (**C**) Western blots depict the expression of LAMP1 and ACTN4 in WT and untreated *GLA*-KO cells and KO cells treated with aGAL, 20 μM clenbuterol, and combined therapy (*n* = 6). (**D**) Lysosomal pH analysis in all conditions demonstrates independent and additive effects of β_2_-adrenergic receptor agonist in *GLA*-KO cells (*n* = 6). (**E**) Lysosomal ROS analysis demonstrates independent and additive effects of β_2_-adrenergic receptor agonist in *GLA*-KO cells (*n* = 6). (**F**) Schematic summary depicting the overall findings of the study: Fabry podocyte lysosomes are characterized by increased size, pH, and ROS production with subsequently decreased function due to Gb3 and SNCA accumulation. This phenotype can be ameliorated through ERT combined with compounds decreasing SNCA accumulation, like β-receptor agonists. Bar graphs depict standard deviation. *P < 0.05, **P < 0.01, ***P < 0.001, ****P < 0.0001. One-way ANOVA with Tukey’s multiple-comparison test.

As SNCA pharmacological modulators are not currently available, we performed connectivity mapping analysis of the transcriptomic profile of Fabry podocytes, which identified the β_2_-adrenergic receptor agonist orciprenaline as the top “anti-Fabry” compound with a relation score of –0.7 (Figure 5A and Supplemental Table 2). Indeed, orciprenaline and another β_2_-adrenergic receptor agonist, clenbuterol, were able to significantly reduce SNCA accumulation in Fabry podocytes (Figure 5B). Furthermore, clenbuterol exhibited a clear dose-dependent effect on SNCA (Supplemental Figure 8, A and B). In accordance with the effects of genetic SNCA reduction, β_2_-adrenergic receptor agonist treatment resulted in decreased lysosomal area (Supplemental Figure 9A) and increased lysosomal acidification in KO podocytes (Supplemental Figure 9B). Strikingly, the combination of ERT and clenbuterol showed an additive effect on the restoration of LAMP1 accumulation (Figure 5C), lysosomal pH (Figure 5D), and ROS production (Figure 5E) in Fabry podocytes, mirroring ultrastructural findings (Supplemental Figure 9C).

## Discussion

The pathomechanisms of FD, specifically Fabry nephropathy, remain incompletely understood owing to several factors. First, the mutational landscape is heterogeneous, as classical hot-spot mutation regions are not commonly observed and no clear genotype-to-phenotype correlations have been described (32). Furthermore, established animal models fail to recapitulate the hallmarks of Fabry nephropathy besides tubular substrate inclusions (24, 25, 33).

In this context, our study contributes to a better understanding of Fabry pathophysiology through the deep phenotyping of a complete *GLA* knockout in podocytes (27) eliminating residual intact and functional enzyme (34, 35). On a functional level, the drastic lysosomal phenotype with decreased acidification, accumulation of ROS, and increased autophagy matches observations made in previous studies and other lysosomal storage diseases (35–37), such as previous reports in primary fibroblasts derived from Gaucher disease patients (38) and endothelial cells exposed to Gb3 (39). However, in contrast to fibroblasts (40), mitochondrial dysfunction did not exert a decisive role in podocytes, indicating that disease- and cell type–specific pathologic pathways may be involved in different lysosomal storage disorders (41). This is also in agreement with the recent finding that podocytes are maintained by anaerobic glycolysis as the predominant metabolic pathway (42).

The primary enzymatic defect and subsequent substrate depositions altered the overall transcriptomic and proteomic landscape of *GLA*-KO podocytes, confirming recent reports (43–46). Interestingly, we observed increased levels of GBA protein, as well as other well-described lysosome-associated proteins such as LIMP2 (encoded by the gene *SCARB2*). The latter has been shown to serve as a specific receptor for glucocerebrosidases and to be involved in proper lysosomal biogenesis (45, 47), suggesting that lysosome impairment extends beyond the initial enzyme defect. In this context, the identification of SNCA as a central player in lysosomal dysfunction resistant to currently available therapies in our cell line, a Fabry mouse model, and patient biopsies reaffirms the existence of an additional mechanism of injury in FD. In a reverse approach, moreover, primary urinary cells of Fabry patients did not demonstrate increased SNCA levels upon Gb3 stimulation, indicating a substrate-independent mechanism as a cause of SNCA accumulation.

SNCA is produced in many cells and constantly degraded through chaperone-mediated autophagy (48) and cathepsin degradation (49). This protein has been implicated in other lysosomal storage diseases (50) and is well known in synucleinopathy-related neurodegenerative diseases such as Parkinson disease (51, 52), in which SNCA is proposed to form a negative-feedback loop that leads to decreased enzymatic degradation (53–55). Importantly, the aggregation of pathological SNCA isoforms has been reported as toxic to cells (56–58), and the modulation of SNCA signaling reverses lysosomal clustering (59), suggesting that SNCA accumulation may represent an intriguing therapeutic target for FD (60).

In agreement with previous reports (13, 14, 61, 62), ERT significantly reduced the volume of podocyte-specific Gb3 inclusion bodies in follow-up biopsies, but without amelioration of podocyte injury and with little effect on glomerular SNCA accumulation. Hence, despite significant reductions in Gb3 accumulation upon ERT and reported correlation of foot process width to Gb3 inclusions (62), remaining Gb3 or substrate-independent mechanisms lead to persistent end-organ damage (63). In this context, our data regarding SNCA accumulation provide a potential explanation to the clinical observations, as they confirm a substrate-independent pathomechanism, and challenge the adequacy of the current standard care for patients with FD.

We believe that our findings have direct clinical implications, as conventional therapy may not be sufficient to prevent and reverse end-organ damage if not initiated very early or accompanied by additional strategies (64, 65). In our study, connectivity mapping identified β-adrenoreceptor agonists as potential FD modulators (66, 67). Interestingly, it has been reported that β_2_-agonists decrease the risk of Parkinson disease via epigenetic downregulation of SNCA gene transcription and protein reduction (68), and β_2_-adrenoreceptor signaling was previously described in podocytes (69). We confirmed the positive effects of β_2_-agonist treatment on lysosomal size, function, and cellular SNCA levels, mirroring the effects of siRNA-mediated SNCA reduction. To our knowledge, this is the first report to delineate a positive effect by targeting of pathways outside the sphingolipid metabolism in Fabry podocytopathy. However, it should be noted that β_2_-agonists will have limited therapeutic potential in FD, as Fabry cardiomyopathy renders patients prone to arrhythmias, which could be potentiated through β_2_-agonist treatment. Nevertheless, the pharmacological amelioration of lysosomal dysfunction through SNCA reduction serves as an ideal proof of concept, indicating that novel strategies could serve as additional therapies, and future studies might also elucidate non-arrhythmogenic downstream signals of β_2_-adrenoreceptor signaling controlling SNCA levels (70).

The observed cell type–specific accumulation of SNCA in the kidneys of Fabry patients should be a further indicator to investigate shared pathomechanisms in lysosomal storage diseases. Notably, a subset of Gaucher disease patients develops Parkinson disease–like conditions mainly due to cellular SNCA accumulation (71), which has been attributed to decreased autophagosomal flux, defective mitophagy, and impaired lysosomal function due to *GBA* mutations (50, 72–74). First evidence has been provided through both a survey and an observational study drawing a link between Fabry and Parkinson disease (60, 75). Similarly, *Gla*-KO mice present with SNCA accumulation in the pons region of the brain in later stages of life (76). Future work may identify to what extent lysosomal dysfunction results in organ-specific phenotypes in different lysosomal storage diseases.

It is possible that additional mechanisms in Fabry podocytopathy remain uncovered, as podocytes in culture tend to partially lose their in vivo expression profile (77). However, the clear accumulation of SNCA deposits detected in glomeruli of Fabry patients depicts the translational aspects of our cell culture model, and the remaining glomerular SNCA deposits after years of therapy provide an explanation why already existing glomerular damage cannot be reversed but only its progression slowed (9–11). While β_2_-agonist drugs, with their pro-arrhythmogenic potential, will not be a therapy choice in multi-organ-affected Fabry patients, early conventional therapy initiation to prevent further accumulation of Gb3 and concomitant cell/organ dysfunction will remain of key importance (78). However, as proven by this study, the complementation with pathway-targeted therapeutic strategies will have the potential to significantly improve long-term outcomes of our patients.

In conclusion, our study systematically maps the signaling networks of Fabry-associated podocyte disease, identifies the role of SNCA in lysosomal impairment and disease severity in Fabry disease, and conceptually proposes an additive pharmacological targeting strategy aiming to halt and reverse Fabry nephropathy.

## Methods

### Quantification of Gb3 deposition and foot process width in renal biopsies.

The subjects were 8 patients (7 male, 1 female) with classic Fabry disease. Fabry disease was confirmed by measurement of leukocyte aGAL activity and/or *GLA* sequencing. Studies were performed in accordance with principles of the Declaration of Helsinki and were approved by the Institutional Review Boards of the University of Washington and the University of Minnesota and by the Regional Ethics Committee of Western Norway. Written informed consent was obtained from each subject.

Renal biopsies were performed as part of a clinical trial protocol (ClinicalTrials.gov NCT00196716) or standard of care before the initiation of ERT and 11 (*n* = 6) or 12 (*n* = 2) months after treatment with agalsidase beta (1 mg/kg every other week). Kidney biopsies from diseased transplant donors obtained before organ removal were studied as controls. Semi-thin sections of 2.5% glutaraldehyde–fixed, plastic-embedded tissues were stained with toluidine blue for identification of glomeruli. Random glomerular sections were prepared for stereological studies as described elsewhere (62). Overlapping digital low-magnification (about ×8,000) images of entire glomerular profiles and high-magnification (about ×30,000) images of glomeruli according to a systematic uniform random sampling protocol were obtained using a JEOL 1010 electron microscope (61). Volume of GL-3 inclusions per podocyte was estimated using a combination of point counting and point-sampled intercept method (21, 62). Podocyte average foot process width was estimated as the reciprocal of slit-length density as previously described (79).

### Cell culture.

Conditionally immortalized human podocytes were provided by M. Saleem (University of Bristol, Bristol, UK). Cells were allowed to proliferate at 33°C in RPMI 1640 medium supplemented with 10% FCS, penicillin/streptomycin, insulin, transferrin, selen, and nonessential amino acids. To induce differentiation, 70% confluent podocytes were switched to 37°C for 10–14 days (unless indicated otherwise). Cells were allowed to differentiate over collagen IV–coated plates (50 ng/μL; Sigma-Aldrich) in all experiments. Rescue experiments were performed by addition of 20 μg/mL α-galactosidase A (aGAL; Sanofi) to the complete medium. aGAL treatment was applied for 96 hours, with 24-hour intervals of enzyme renewal, to cells differentiated for 10 days. Venglustat was administered at 300 nM concentration for 120 hours, with 24-hour intervals of compound renewal, to cells differentiated for 8 days. The β_2_-adrenergic receptor agonists clenbuterol hydrochloride (European Pharmacopoeia) and orciprenaline (Sigma-Aldrich) were used in our experiments. Different concentrations were applied for 96 hours (as indicated in the figure legends). β_2_-Adrenergic receptor agonist treatment was applied to 10-day-differentiated cells and renewed every 24 hours for 96 hours. Primary urinary cells were collected from Fabry patients in the International Center for Lysosomal Storage Disorders (ICLD; Hamburg, Germany) according to Ethics Statement PV3501 approved by the Ethics Board of the Board of Physicians, Hamburg. Cells were taken into culture and expanded as described previously (45, 80). After reaching sufficient confluence, cells were treated with normal proliferation medium (80) supplemented with DMSO (vehicle) or 100 nM lyso-Gb3 (Sigma-Aldrich) in DMSO for 48 hours.

### Generation of isogenic GLA-KO human podocytes.

CRISPR/Cas9 genome editing was applied to generate *GLA*-KO podocytes in vitro as previously described (81). Briefly, CRISPR/Cas9 genome editing with 2 different gRNAs [gRNA1: 5′-TTGTCCAGTGCTCTAGCCCC(AGG)-3′; gRNA2: 5′-CAGTGCAGCCAGCCCATGGT(AGG)-3′] targeting the first exon of the human *GLA* gene was used. A Web-based platform was operated in order to design these gRNAs (E-CRISP, http://www.e-crisp.org/E-CRISP/). The gRNAs being subcloned in targeting CRISPR nuclease vectors with orange fluorescent protein (GeneArt Life Technologies) according to the manufacturer’s protocol were inserted in the immortalized human podocytes via electroporation. Mixed cell populations were validated with restriction enzyme digestion with NcoI (5′-C||CATGG-3′; New England BioLabs Inc.) for gRNA2 or with an indel mutation detecting and cleaving the enzyme (Genomic Cleavage Detection Kit, GeneArt Life Technologies) for gRNA1. After an incubation period of 48 hours, single cells were selected via FACS, sorted into 96-well plates, and further expanded into isogenetic clone colonies. The DNA sequences were analyzed using the Sanger technique after initial DNA isolation and amplification (forward primer: 5′-TGGAAATAGGGCGGGTCAAT-3′; reverse primer: 5′-TTCCCCAAACACACCCAAAC-3′). The sequencing results proved the sex of the clone cell line used to be male. Hemizygous *GLA*-mutated clones translated in silico were reviewed for frameshift mutations and premature stop codons.

### Electron microscopy of cell culture podocytes.

Cell samples were fixed in 4% paraformaldehyde plus 1% glutaraldehyde in 0.1 M phosphate buffer overnight. After contrasting using 1% osmium tetroxide in 0.1 M phosphate buffer (45 minutes at room temperature) and 1% uranyl acetate (in 70% ethanol, room temperature), samples were dehydrated in an ascending ethanol series and embedded in epoxy resin (Durcupan, Sigma-Aldrich). Ultrathin sections of approximately 70 nm thickness were prepared using a Leica Ultracut UC6. For imaging, a Philips CM100 transmission electron microscope was used.

### RNA sequencing.

Conditionally immortalized human podocyte WT and CRISPR/Cas9 *GLA*-KO cell lines were differentiated at 37°C at 70% confluence for 10–14 days. Total RNA of cells was isolated using the phenol/chloroform method as previously described (82).

Library preparation and sequencing were performed by GATC Biotech. All raw data were deposited in the NCBI’s Gene Expression Omnibus database (GEO GSE186258).

### Connectivity mapping.

The top 100 up- and downregulated genes from RNA sequencing data were uploaded to the next-generation connectivity mapping tool CLUE (67). Output analysis and ranking were automatically performed by the CLUE website.

### Proteomics analysis.

In order to identify new regulators that may play a role in the lysosomal dysfunction, we studied the whole proteome in *GLA*-KO cells by using quantitative proteomics based on stable isotope labeling by amino acids in cell culture (SILAC) and our podocyte Fabry model and by analyzing 3 Fabry clones versus 3 WT clones. For mass spectrometry (MS) analysis, SILAC labeling of human immortalized podocytes was performed for 14 days as previously described (83). Based on SILAC labeling and protein concentration, WT and KO samples were mixed 1:1 for MS analysis. Liquid chromatography–tandem MS (LC-MS/MS) data analysis was performed as reported previously (84). The MS raw data files were uploaded into MaxQuant software version 1.4.1, which performs peak and SILAC pair detection and generates peak lists of mass error–corrected peptides and database searches. We identified nearly 2,300 proteins, among which 321 are lysosome-enriched proteins. The top 10 up- and downregulated were considered as targets of high interest for further functional analysis. All raw data and original result files were deposited to the ProteomeXchange Consortium (http://proteomecentral.proteomexchange.org) via the PRIDE partner repository with the data set identifier PXD029618.

### Building GLA-specific network modules.

Network-based approaches are based on the assumption that proteins that participate in the same biological processes or share molecular functions are not scattered randomly but tend to cluster and build functionally relevant modules within the human interactome. In order to identify the disease module that connects the detected up- and downregulated proteins associated with *GLA* knockout, we used a large data set of known protein interactions recently compiled by Cheng and colleagues (85). It consists of 16,677 proteins (nodes) connected by 243,603 protein interactions (edges). One way to build the module would be to search for known direct interaction partners of the up- and downregulated proteins (seed proteins), calculate the size of the resulting largest connected module between them, and evaluate whether this size significantly differs from random expectation. However, this method would favor highly connected proteins in the human interactome. In order to detect connections between the seed proteins in an unbiased way, we used a recently published disease module detection algorithm (DIAMOnD). DIAMOnD takes into account the “connectivity significance” of proteins to a set of seed proteins and helps to build a module around them (86). The algorithm calculates the probability that a protein with *k* links has exactly *k_s_* links to the seed proteins using the hypergeometric distribution and determines the *P* value that it has more connections to the seed proteins than expected. The protein with the lowest *P* value is then added to the module, and a new iteration starts. Since the algorithm could iterate over all 16,677 proteins of the interactome, a break-off criterion to determine the final optimal module size needs to be defined.

### Determining the final module size.

In order to determine the final module size, we used the method that was recently described by Halu and colleagues (87). DIAMOnD ranks the proteins that are not seed proteins according to their *P* value (see above) and incorporates the protein with the lowest *P* value into the module. After each iteration, the resulting module size, i.e., the number of nodes that are directly connected to each other, is determined and compared against random expectation (1,000 random network samples), resulting in a respective *z* score: *z* = (*module* – *randommodule*)/σ*random*, where *module* and *randommodule* are the sizes of the resulting largest connected module and the random expectation, respectively, and σ*random* is the standard deviation of the calculated 1,000 random module sizes. After each iteration, we determined how many seed proteins were integrated into the module. As proposed by Halu et al., the module size where all seed proteins were integrated into the module and where the corresponding *z* score was above 1.96 (significant *z* score) was taken as the final module size.

### aGAL enzyme activity measurement.

The fluorometric method of measuring the enzyme activity of aGAL with 4-methylumbelliferyl-α-d-galactopyranoside has previously been fully described (88–90). In addition, *N*-acetylgalactosamine has been shown to significantly inhibit α-galactosidase B activity (90). Briefly, cells from several *GLA*-KO isogenetic clones and WT cells were counted and pelleted. The pellets were lysed in 500 μL lysis buffer (27 mmol/L sodium citrate, 46 mmol/L sodium phosphate dibasic, 0.1% Triton X-100, 1 M HCl, in ddH_2_O, pH 4.6) by pipetting on ice. Proteins were then separated by centrifuging at 13.2*g*, and the protein concentration was determined via the Pierce BCA protein assay. Ten microliters of each sample with 3 technical replicates was incubated with 25 μL test buffer (27 mmol/L sodium citrate, 46 mmol/L sodium phosphate dibasic, 6 mmol/L 4-methylumbelliferyl-α-d-galactopyranoside, 90 mmol/L *N*-acetyl-d-galactosamine, 1 M HCl, in ddH_2_O, pH 4.6) at 37°C for 1, 6, or 11 hours. Subsequently, 35 μL stop buffer (0.4 mol/L glycine, 5 N NaOH, in ddH_2_O, pH 10.8) was added and the fluorescence measured. After initial shaking with amplitude of 1 mm for 10 seconds and a 25-time excitation with 355 nm, the emissions of 455 nm were measured over an integration period of 20 microseconds. A standard activity curve was established using aGAL from green coffee beans (Sigma-Aldrich) with known aGAL activity in serial dilution.

### MS measurement of Gb3.

Differentiated WT and KO podocytes were treated for 48 hours with α-galactosidase or PBS for MS quantification of Gb3 as described previously (91). By the end of incubation, cells were washed twice with PBS, harvested by trypsin, and washed again with ice-cold PBS and directly lysed in 0.2% Triton-PBS. Equal volume was taken from each sample for total protein measurement in order to normalize the final readings (Pierce BCA protein assay). Lysate was then stored at –20°C, and Gb3 quantification was performed by pharm-analyt Labor GmbH.

### Lyso-Gb3 solid-phase extraction.

For solid-phase extraction, 40 μL of the plasma sample was diluted in 320 μL water/acetonitrile/MeOH (final 1:2:2) and 36 μL 1 M HCl in a 1.5 mL reaction tube. Cells were resuspended in 360 μL of water/acetonitrile/MeOH (1:2:2), followed by homogenization and lysis at –20°C for 2 hours. For solid-phase extraction, Oasis MCX 1 cc Vac cartridges, 30 mg Sorbent, 60 μm (Waters Corp.) were used along with a vacuum manifold and pump. The cartridges were activated with 1,000 μL MeOH 100% and then 1,000 μL 1 M HCl. Then the samples were loaded. Columns were washed with 1,000 μL HPLC-grade water plus 2% formic acid and afterward with 1,000 μL MeOH plus 0.2% formic acid. The lipids were eluted with 600 μL MeOH plus 2% NH_4_OH, and excess solvent was evaporated (CentriVap Concentrator, Labconco) at 10°C. After evaporation, the eluate pellets were resuspended in 50 μL 1:1 acetonitrile and water plus 0.2% formic acid and centrifuged at 18,000*g*, 4°C, for 10 minutes, and the supernatant was transferred into analytical vials for the LC-MS measurement.

### Lyso-Gb3 LC-MS analysis.

Targeted metabolomics analysis was performed on a triple-quadrupole (Agilent Triple Quadrupole 6495C) coupled with an ultra-high-pressure liquid chromatography system (1290 Infinity, Agilent Technologies). Data acquisition was done with Agilent MassHunter Workstation Data Acquisition (version 10.1). Samples were separated on a BEH amide column (1.7 μm, 2.1 × 100 mm) (Waters A/S) with buffer A (HPLC-grade water with 20 mM ammonium formate plus 0.1% formic acid) and buffer B (acetonitrile plus 0.1% formic acid). For separation, the following gradients (A/B) were used with a flow rate of 0.4 mL/minutes: T0, 10/90; T1, 35/65; T4, 35/65; T6, 50/50; T8.5, 10/90; and T12.5, 10/90. Two microliters of the sample was injected each run. Multi-reaction monitoring was used as the scan type. The transition list and retention times for measured lyso-Gb3 were: 786.45 → 282.2 (quantifier), 786.45 → 264.2 (qualifier). The collision energies (MS2 or quantifier and qualifier ion transitions) were optimized. Electrospray ionization source parameters were: gas temperature = 180°C, gas flow = 12 L/min, nebulizer = 20 psi, sheath gas temperature = 200°C, sheath gas flow = 12 L/min, cap voltage = 4,000 V, nozzle voltage = 1,500 V.

### Dual-emission ratiometric measurement of lysosomal pH and LysoTracker.

LysoSensor Yellow/Blue DND-160 (Life Technologies) was used according to the manufacturer description in order to determine the lysosomal acidity in cultured cells. Briefly, cells were suspended and labeled with 10 μM LysoSensor DND-160 for 2 hours at 37°C in 10% medium; cells were then pelleted and washed in PBS twice. The labeled cells were treated for 10 minutes with 10 mM monensin (Sigma-Aldrich) and 10 M nigericin (Sigma-Aldrich) in 25 mM 2-(*N*-morpholino)ethanesulfonic acid (MES) calibration buffer, pH 3.5–8.0, containing 5 mM NaCl, 115 mM KCl, and 1.2 mM MgSO_4_. Cells were then distributed in black 96-well plates (2,500 cells per well), and the fluorescence was measured with a Tecan plate reader. Light emitted at 440 and 535 nm in response to excitation at 340 and 380 nm was measured, respectively. The ratio of light emitted with 340 and 380 nm excitation was plotted against the pH values in MES buffer, and the pH calibration curve for the fluorescence probe was generated (92). In order to study the lysosomal structure and to score the cellular lysosomal mass, LysoTracker Red DND-99 (Life Technologies) was used according to the manufacturer’s recommendation. LysoTracker was applied to differentiated podocytes for 30 minutes at 37°C with a final working concentration of 50 nM. Cells were washed, and images were taken using a Zeiss Axio Observer microscope, equipped with a ×63 objective and Apotome function, and analyzed using CellProfiler (https://cellprofiler.org/). The lysosomal fraction was blotted as a percentage of lysosomal to cellular area.

### Oxidative stress detection.

DCFDA Assay (Thermo Fisher Scientific) was used to measure ROS level in adherent cells (93). Differentiated cells were harvested and seeded back in a dark, clear-bottom 96-well microplate with a concentration of 2,500 cells per well and allowed to adhere overnight. Cells were then washed with PBS and incubated with DCFDA solution (20 μmol in PBS supplemented with Ca^2+^ and Mg^2+^) by adding 100 μL/well for 45 minutes at 37°C in the dark. DCFDA solution was removed, wells were washed twice with PBS, and the fluorescence was immediately measured by Tecan reader (excitation/emission = 485/535 nm).

### Thin-layer chromatography.

Lipid extraction was carried out in principle according to the method of Bligh and Dyer (94). Pelleted cells were lysed by osmotic shock using ddH_2_O, then transferred into a glass tube and mixed and vortexed with chloroform/methanol (1:2) for 1 minute. Then 1.25 mL of chloroform and 1.25 mL of ddH_2_O were added and mixed for 15 seconds. For phase separation, the solution was centrifuged at 1,700*g* for 10 minutes at 15°C. The organic phase was transferred to a new glass tube. The hydroalcoholic phase was washed once with 1.5 mL of chloroform, mixed for 15 seconds, and then centrifuged at 3,000*g* for 10 minutes at 15°C. The organic phases were combined, and the solvent was evaporated under a stream of nitrogen. Dried lipids were either stored under an argon atmosphere or resolved in chloroform/methanol (2:1) for thin-layer chromatography (TLC). Lipid extracts were loaded on 2 silica plates and separated by TLC using methanol/chloroform/ddH_2_O (60:35:8) as a mobile phase. Afterward, 1 silica plate was incubated with *p*-anisaldehyde/acidic alcohol solution and placed in an oven for 15–30 minutes at 120°C to stain overall lipids. For specific detection of Gb3, the other plate was fixed by 3.75% (wt/vol) polyisobutylmethacrylate/*n*-hexane, blocked with 1% (wt/vol) BSA/PBS (containing calcium and magnesium), and incubated afterward with biotinylated Shiga toxin B subunit (1.8 μg/mL) and subsequently with alkaline phosphatase–conjugated streptavidin (2 μg/mL). Gb3 bands were visualized by a colorimetric reaction with nitroblue tetrazolium/BCIP substrate solution (Thermo Fisher Scientific). The chromatogram was densitometrically analyzed and documented using Fusion FX (Vilber Lourmat) and ImageJ software (NIH).

### Seahorse XFp mitochondrial analysis and ATP measurement.

Optimization of cell density for human podocytes of the respective genotype as well as optimization of the working concentration titers for each individual inhibitor was conducted prior to the Seahorse XFp experiments according to the manufacturer’s instructions (Agilent Technologies). Human podocytes were seeded at a density of 15,000 cells per well. The Seahorse XFp Mito Stress Test was performed following the manufacturer’s instruction. Specifically, podocytes were seeded on XFp microplates 24 hours before the experiment. On the day of the assay, cells were rinsed and XF assay buffer was added for further equilibration. Afterward the plate was incubated for 1 hour at 37°C in a non-CO_2_ incubator. All medium and solutions of mitochondrial complex inhibitors were adjusted to pH 7.4 before each assay. Following 4 baseline measurements of oxygen consumption rate (OCR) and extracellular acidification rate (ECAR), 4 Mito Stress Test inhibitors of the respiratory chain were sequentially injected into each well. Three OCR and ECAR readings were taken after addition of each inhibitor and before automated injection of the subsequent inhibitor. Mitochondrial complex inhibitors, in order of injection, included oligomycin (1.5 μM) to inhibit complex V, FCCP (1.0 μM) to uncouple the proton gradient, antimycin A (1.0 μM; inhibitor of complex III), and rotenone (1.0 μM; complex I inhibitor). OCR and ECAR were automatically calculated by Seahorse XFp software version 2.2.0 (Seahorse Bioscience). After each experiment, podocytes were fixed with paraformaldehyde and nuclei were stained with DAPI. Olympus ScanR Screening Station for high-throughput microscopy detection was used for assessment of cell number to normalize XFp analysis data.

### SNCA knockdown and overexpression in immortalized human podocytes.

Generation of siRNA-mediated knockdown of *SNCA* in immortalized human podocytes was performed using the following sequences: T1-1: 5′-CAUAGUCAUUUCUAAAAGUUU-3′; T2-1: 5′-GGAUUUAUGUGGAUACAAAUU-3′. These sequences have been previously tested and published (95). Transfections were performed using Amaxa nucleofector technology (Lonza) according to the manufacturer’s instructions. Cells were allowed to differentiate for 10 days before transfection and seeded back for 48 or 72 hours before analysis. The knockdown efficiency was compared with scramble transfection using Western blotting 48 and 72 hours after transfection.

### Western blot and immunofluorescence.

The buffers, the systems, and the protocols that were applied in Western blotting can be found in our previous publication (96). The following antibodies were used in Western blot experiments: α-galactosidase (OriGene Technologies, TA336243), α-tubulin (Sigma-Aldrich, T9026), actinin 4 (Abcam, ab108198), p62 (Cell Signaling Technology, 5114), LC3 (Invitrogen, PA1-16931), SNCA (Santa Cruz Biotechnology, sc-12767), SCARB2 (Lifespan Biosciences, LS-B3225-0.05), TTYH3 (Origene, TA339554), MFEG8 (Sigma-Aldrich, HPA002807), CD63 (Proteintech, 25682-1-AP), DDP (Novus Biologicals, H00001678), PLSCR3 (Abcam, ab57554), GBA (Abcam, ab96246), and ITM2B (Abcam, ab119044). TOM20 (Santa Cruz Biotechnology, sc-11415) and LAMP1 (Abcam, ab24170) were used for immunofluorescence staining. Immunofluorescence staining was performed on differentiated cells seeded in collagen-coated IV 8-well chamber slides (Ibidi). Cells were fixed in 4% paraformaldehyde in PBS for 10 minutes and permeabilized using 0.1% Triton X-100 in PBS for TOM20 and methanol for LAMP1. Permeabilized cells were washed in PBS and blocked with 5% BSA in PBS for 1 hour at room temperature. Primary and secondary antibodies were diluted in blocking solution and incubated for 120 minutes and 45 minutes, respectively. *Z*-stack images of TOM20- and LAMP1-stained cells were acquired and presented as maximum-intensity projections in respective figures.

### Immunostaining of SNCA.

Kidney biopsies before the onset of enzyme replacement therapy and after 5 years of treatment were taken from a selection of a larger patient series already described elsewhere (97). Paraffin sections of 3 μm thickness were cut and incubated at 42°C overnight. Sections were then deparaffinized and rehydrated (20 minutes in xylene, 10 minutes in 100% ethanol, and 5 minutes in 95%, 85%, 75%, and 50% ethanol). Slides were washed for 5 minutes in 1× PBS/0.1% Tween. Antigen retrieval solution (pH 6) was used in a steam cooker for 30 minutes, and the slides were allowed to cool for 20 minutes on ice. Slides were washed 3 times with PBS and blocked with 5% BSA in PBS for 45 minutes. Endogenous peroxidase was inactivated with DAKO peroxidase inhibitor for 15 minutes. Primary antibody — SNCA (Santa Cruz Biotechnology, sc-12767) — was diluted in blocking solution (1:200), and the samples were incubated for 2 hours at room temperature. Goat anti-rabbit biotinylated (1:500; DAKO) was added for 30 minutes and streptavidin/HRP (1:500; DAKO) for 30 minutes followed by ACE substrate (DAKO) for 10 minutes. Slides were washed after every step with 1× PBS. Finally, slides were mounted using permanent mounting medium. Stained slides were scanned with the Aperio ScanScope XT system (Leica Biosystems Imaging) at ×40 objective magnification and viewed in eSlide Manager (Leica Biosystems Imaging). Staining intensities of SNCA were analyzed with the color deconvolution method (98). Percentage total positive pixel count was acquired with the Aperio Color Deconvolution algorithm v9 (Leica Biosystems Imaging) from annotated glomerular regions.

### In-cell Western blotting for SNCA.

Primary urinary cells were grown in a 96-well plate coated with 0.1% Gelatine (MilliporeSigma) until confluent. After treatment with vehicle/lyso-Gb3, cells were washed in PBS, fixed in 4% paraformaldehyde, washed again in PBS, and blocked/permeabilized in 5% BSA, 0.1% Triton X-100 in PBS for 1 hour at room temperature. After 3 washing steps in PBS, cells were stained with primary antibody (1:200; Sigma-Aldrich, HPA005459) overnight. The next day, after 3 additional washing steps in PBS, cells were incubated in secondary antibody (1:200; LI-COR IRDye 800cw) and Draq5 nuclear stain (1:1,000; Abcam, 108410) for 1 hour at room temperature. Cells were washed again 3 times and imaged with a LI-COR Odyssey imager at 3 μm focus, 89 μm resolution.

### Mouse line and in vivo treatment.

hR301Q α-galactosidase A Tg/KO mice (Tg/KO) were used as a model of Fabry disease (99). Specifically, these mice are homozygous for endogenous *Gla* knockout and express the human transgene *GLA* carrying the R301Q mutation under the transcriptional control of the human GLA promoter. Six-month-old homozygous Tg/KO mice were treated with 1-deoxygalactonojirimycin (migalastat, Amicus Therapeutics) or with ibiglustat (Venglustat, Cayman Chemical Co.) at the same dose of 100 mg/kg/d (5 mice per group). Both migalastat and Venglustat were administered orally to mice in drinking water. The appropriate concentration of drugs in drinking water was determined based on the average daily water consumption of Fabry Tg/KO mice (~5 mL/d per mouse), and the solutions were made fresh weekly. Migalastat treatment was administered in alternate months (99), while Venglustat was administrated continuously from 6 to 9 months of age. Control Tg/KO mice and C57BL/6 WT mice were exposed to DMSO (vehicle) in a 1:1,500 dilution in drinking water. At study completion, mice were euthanized, and the organs of interest (heart, kidney, liver, and brain) were quickly removed, rinsed in cold PBS, and immediately frozen in liquid nitrogen for subsequent analysis.

### SNCA ELISA.

Human and murine SNCA were determined using the Human Alpha-synuclein ELISA (Abcam, ab260052) or Mouse Alpha-synuclein ELISA (Abcam, ab282865) according to the manufacturer’s protocols.

### Cathepsin activity assay.

Cathepsin D activity was determined according to Huarcaya et al. (49). In short, cells were lysed in acidic buffer (50 mM sodium acetate, 0.1 M NaCl, 1 mM EDTA, 0.2% Triton X-100, pH 4.5) and 2 μg incubated with 0.1 μM quenched fluorogenic peptide (Enzo, BML-P145) and 0.025 mM leupeptin (Enzo, ALX-260-009-M025) at 37°C for 30 minutes in 100 μL additional lysis buffer. Then samples were measured using 322 nm excitation and 381 nm emission.

### Images.

See supplemental material for full, uncut gels.

### Data availability.

The RNA-Seq and proteome data reported here were deposited in the NCBI’s Gene Expression Omnibus (GEO GSE179975) database and ProteomeXchange Consortium (PXD029618).

### Statistics.

Data are expressed as scatter or violin plots. Unpaired, 2-tailed Student’s *t* test or 1-way ANOVA with Tukey’s multiple-comparison test was used based on data distribution. Statistical significance was defined as *P < 0.05, **P < 0.01, ***P < 0.001, and ****P < 0.0001. The number of independent experiments and the total number of analyzed cells are specified in each figure legend.

### Study approval.

Renal biopsies were performed as part of a clinical trial protocol (ClinicalTrials.gov NCT00196716) or standard of care before the initiation of enzyme replacement therapy. Ethical permission was granted by the ethics committee of the Western Regional Health Authority in Norway (REK Vest 2010/2483). Informed consent was signed by the patient and/or their designees in all cases. Kidney biopsies from the Norwegian Kidney Biopsy Registry with normal light microscopic or electron microscopic appearance served as controls (REK Vest 2013/553). Animal husbandry and all experiments were conducted under Institutional Animal Care and Use Committee–approved protocols at Federico II University.

## Author contributions

TBH initiated this study. FB and AA designed the research study, conducted experiments, acquired data, analyzed data, and wrote the manuscript. FB was chosen as first co–first author for the planning and coordination of the revision process and the revision of the manuscript. DS, MR, MW, OE, NW, JG, SDL, FH, MNW, BD, PR, AM, WS, KVC, SWG, OK, OH, TB, FG, WL, TE, WR, CT, and BN conducted experiments, acquired data, and analyzed data. NM and MM provided resources and acquired patient samples. ACM, KS, AH, and CMS provided resources and revised the manuscript. HPM, CMS, GI, and MMR designed the research study and provided resources. VGP, CS, and TBH designed and supervised the research study, provided resources, and wrote and revised the manuscript.

## Supplementary Material

Supplemental data

Supplemental table 1

Supplemental table 2

## Version 1 04/04/2023

In-Press Preview

## Version 2 06/01/2023

Electronic publication
