# Supplementary material for: Accumulation of α-synuclein mediates podocyte injury in Fabry nephropathy
Source: J Clin Invest. 2023 Jun 1;133(11):e157782. doi: 10.1172/JCI157782 (PMC10232004; doi:10.1172/JCI157782)

Fig. S1

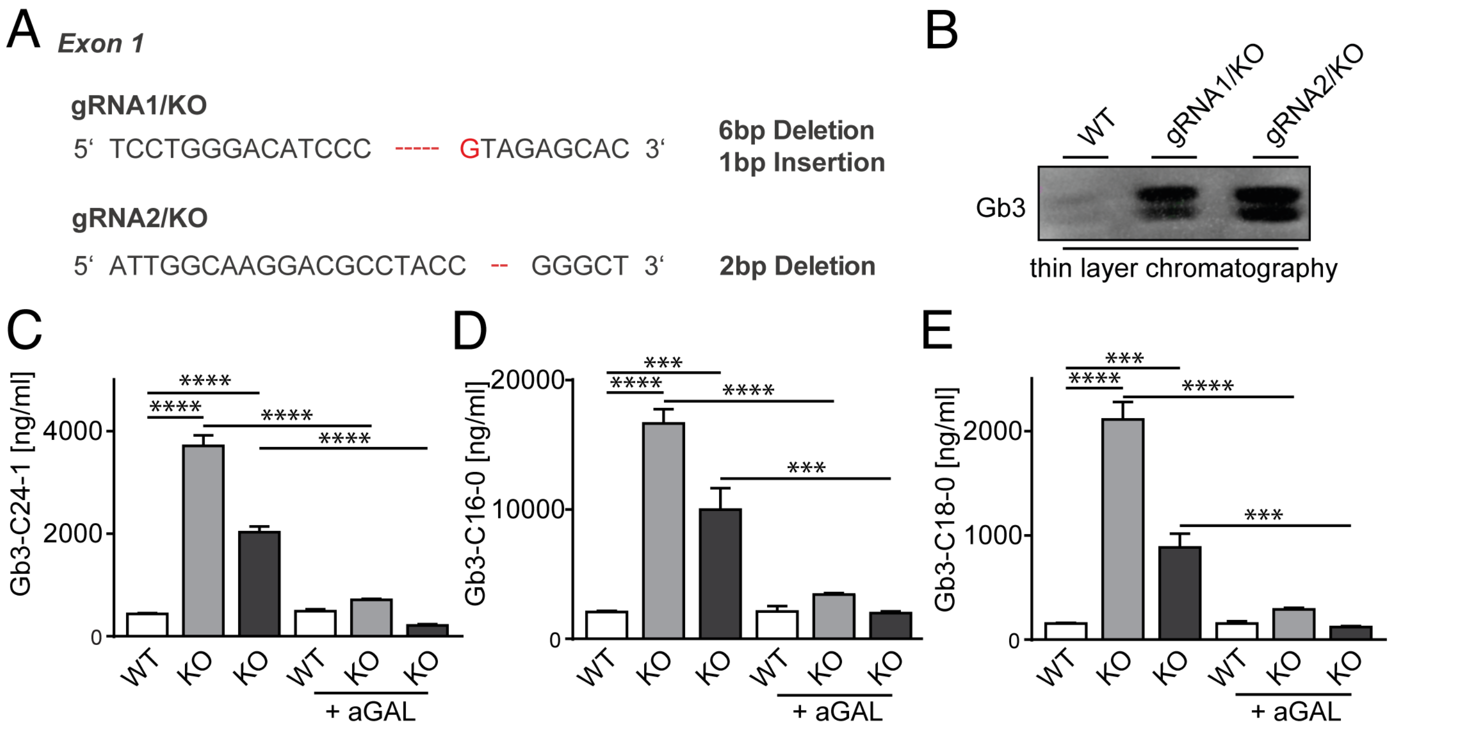

Figure S1:
(A) Two gRNAs were used to target the first exon within the GLA locus inducing premature stop codons. (B) Thin layer chromatography reveals a substantial accumulation of GB3 in the KO clones A and B. (C, D and E respectively) Mass spectrometry analysis of GB3 isoforms shows a significant accumulation of GB3-C24-1, GB3-C16-0 and GB3-C18-0 in GLA KO compared to WT cells. The expression level of these isoforms is nearly normalized upon 96hours of enzyme therapy.

Fig. S2

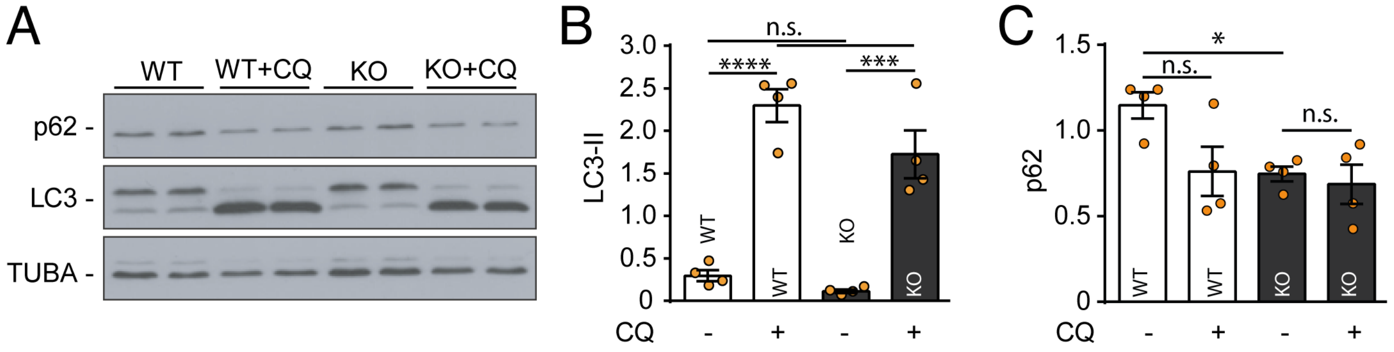

Figure S2:
(A) Western blot shows a comparable LC3-II accumulation and P62 level in GLA KO and WT cells after 2 hours of (100µM) chloroquine treatment. (B) Bands Quantification confirms a significant increase in LC3 in both clones compared to untreated cells but no difference between the genotypes has been noticed. (C) p62 in KO cells is less abundant in KO clones compared to WT cells at basal condition but not different upon chloroquine treatment (n=4).

Fig. S3

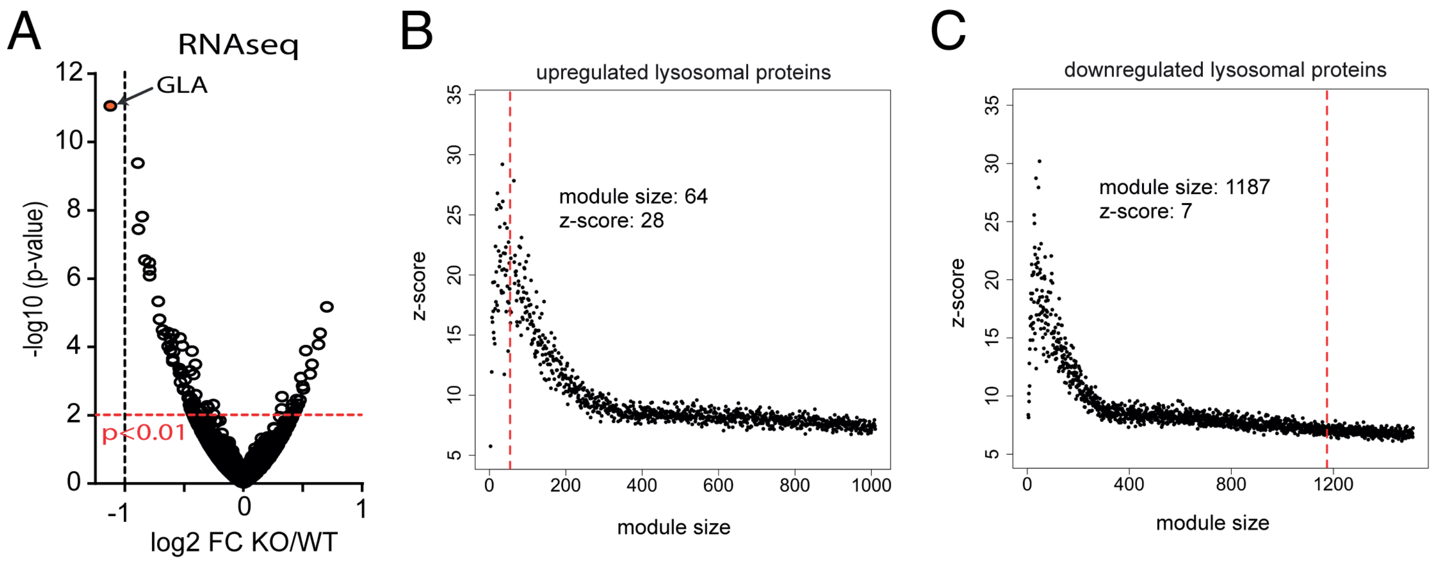

Figure S3:

(A) RNA sequencing of WT and GLA-KO cells where GLA gene was the top downregulated gene (arrow). (B) Determining the module size of upregulated seed proteins. The z-score of the resulting module is plotted against the module size, which is determined by the known protein interactions of the DIAMOnD proteins and upregulated seed proteins with GLA. The red dashed line indicates the module size (number of genes n=64) where all upregulated seed proteins are integrated into the module. The resulting module has a significantly larger size than expected by random (z-score = 28). (C) Determining the module size of downregulated seed proteins. The z-score of the resulting module size is plotted against the module size, which is determined by the known protein interactions of the DIAMOnD proteins and downregulated seed proteins. The red dashed line indicates the module size (number of genes n=1185) where all downregulated seed proteins are integrated into the module. The resulting module has a significantly larger size than expected by random (z-score = 7).

Fig. S4

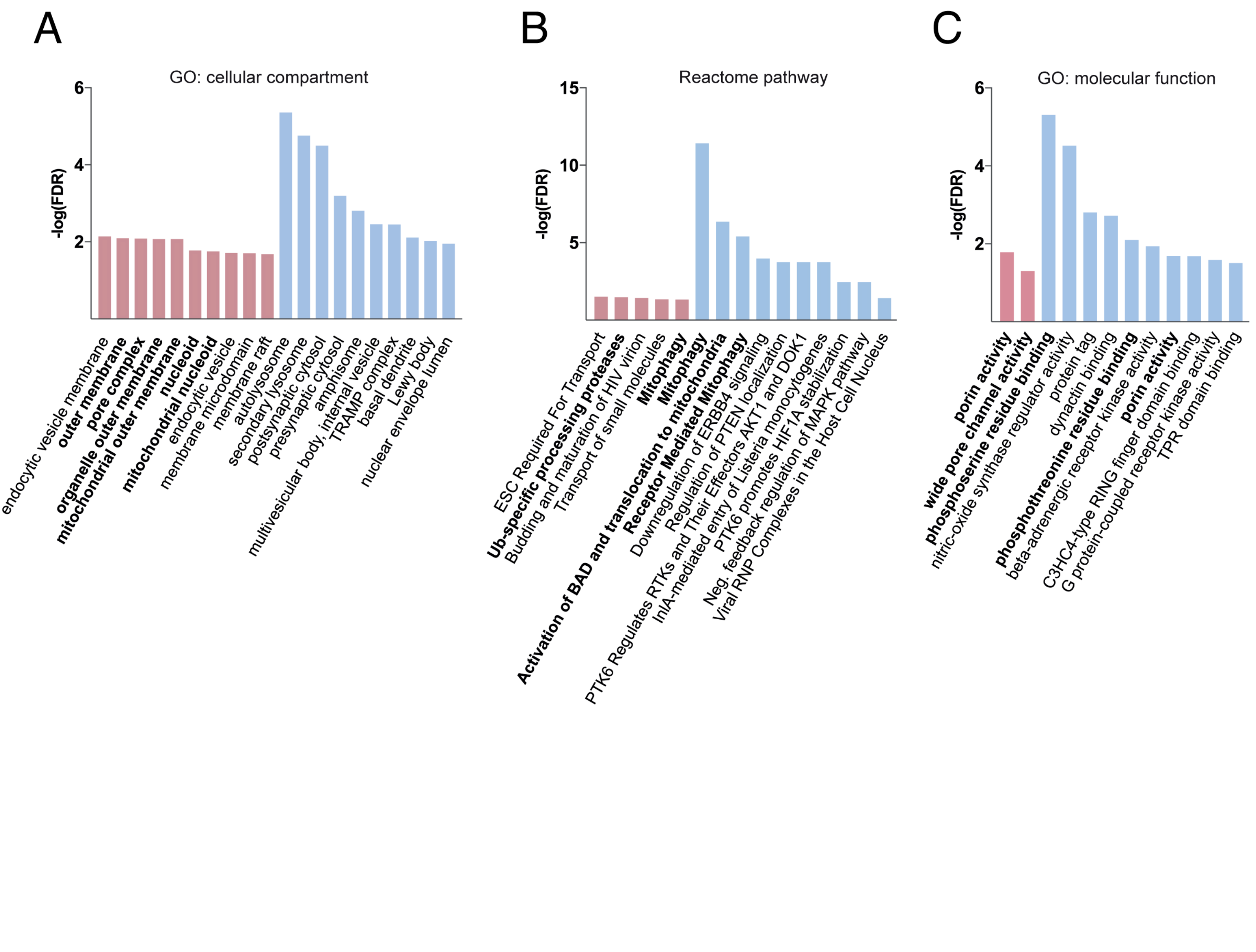

Figure S4:
Overrepresented terms for the upregulated seed protein network (red) and downregulated seed protein network (blue) through (A) GO: cellular compartment, (B) Reactome pathway and (C) GO: molecular function annotation. FDR values were retrieved using the panther webtool (http://www.pantherdb.org, as of May 2019).

Fig. S5

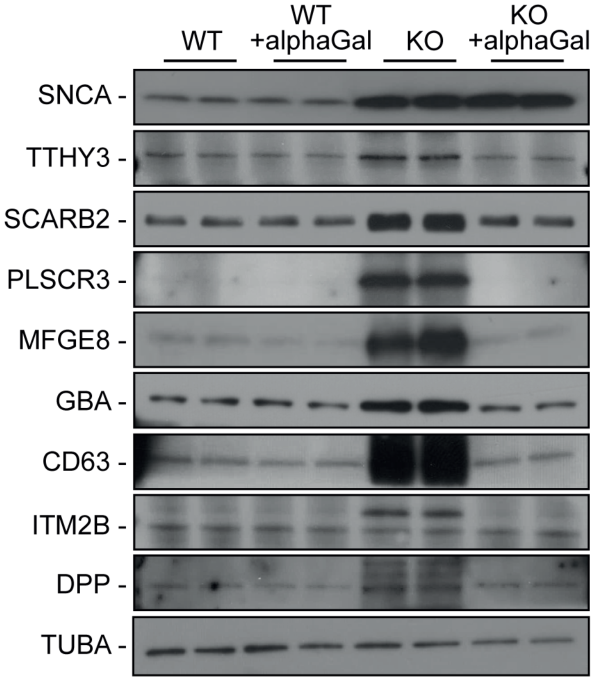

Figure S5:
Western blots confirming the over-expression of the top ten up regulated proteins) reversible upon 96hours of aGAL therapy. SNCA and Tubulin A blot also displayed in Figure 3B.

Fig. S6

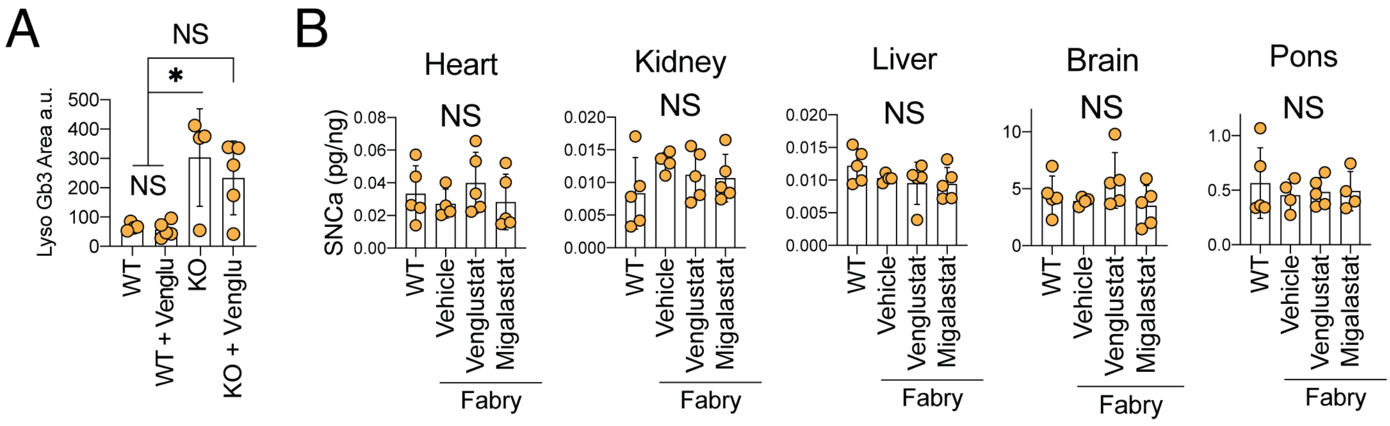

Figure S6:
(A) Mass spectrometry analysis of Lyso-GB3 shows a significant accumulation of in GLA KO compared to WT cells with a non-significant modulation of Lyso-Gb3 levels in Venglustat treated cells (n=5), (B) Mouse SNCA ELISA depicting no significant differences in SNCA accumulation between 9 month old hR301Q mice in different organs without a significant effect of treatment with Venglustat or Migalastat for 3 months (n≥4).

Fig. S7

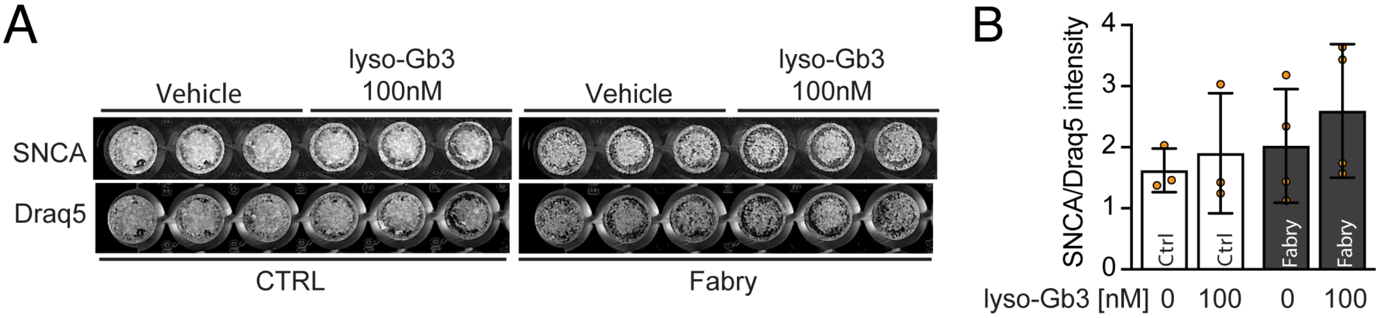

Figure S7:
(A & B) The quantification of in-cell western blot of SNCA vs Draq5 showing no significant differences between groups or upon Gb3 treatment (n=3 controls and 4 Fabry).

Fig. S8

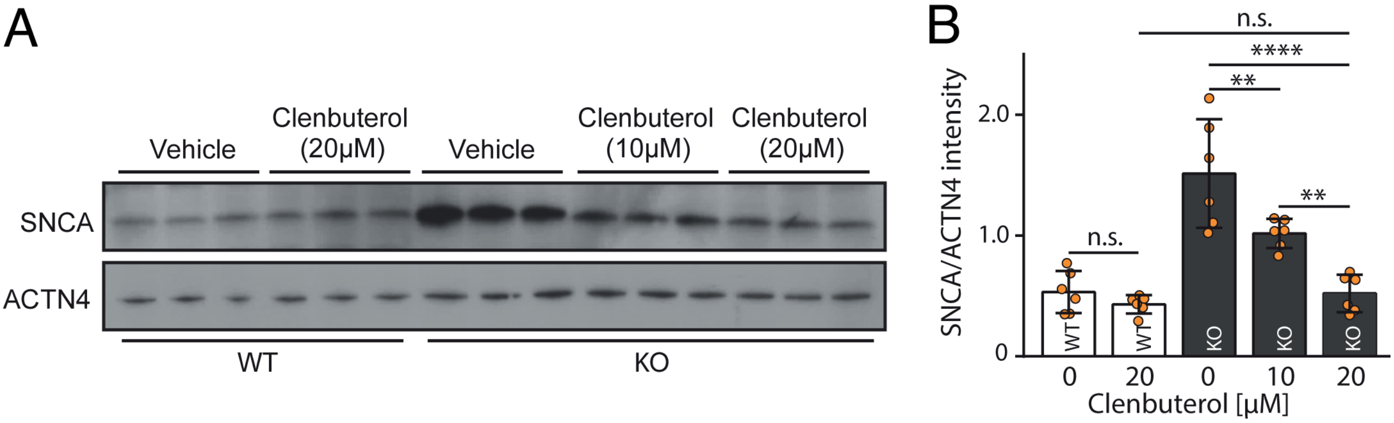

Figure S8:
(A) Western blots of SNCA and ACTN4 in WT vehicle-treated cells, KO vehicle-treated cells, KO 10µMClenbuterol-treated cells and KO cells treated with 20 µM Clenbuterol (B) The quantification of western blot bands of SNCA vs ACTNn4 showing a Clenbuterol dose dependent inhibition of SNCA (10 µM and 20 µM for 96 hours, N=6 per group).

Fig. S9

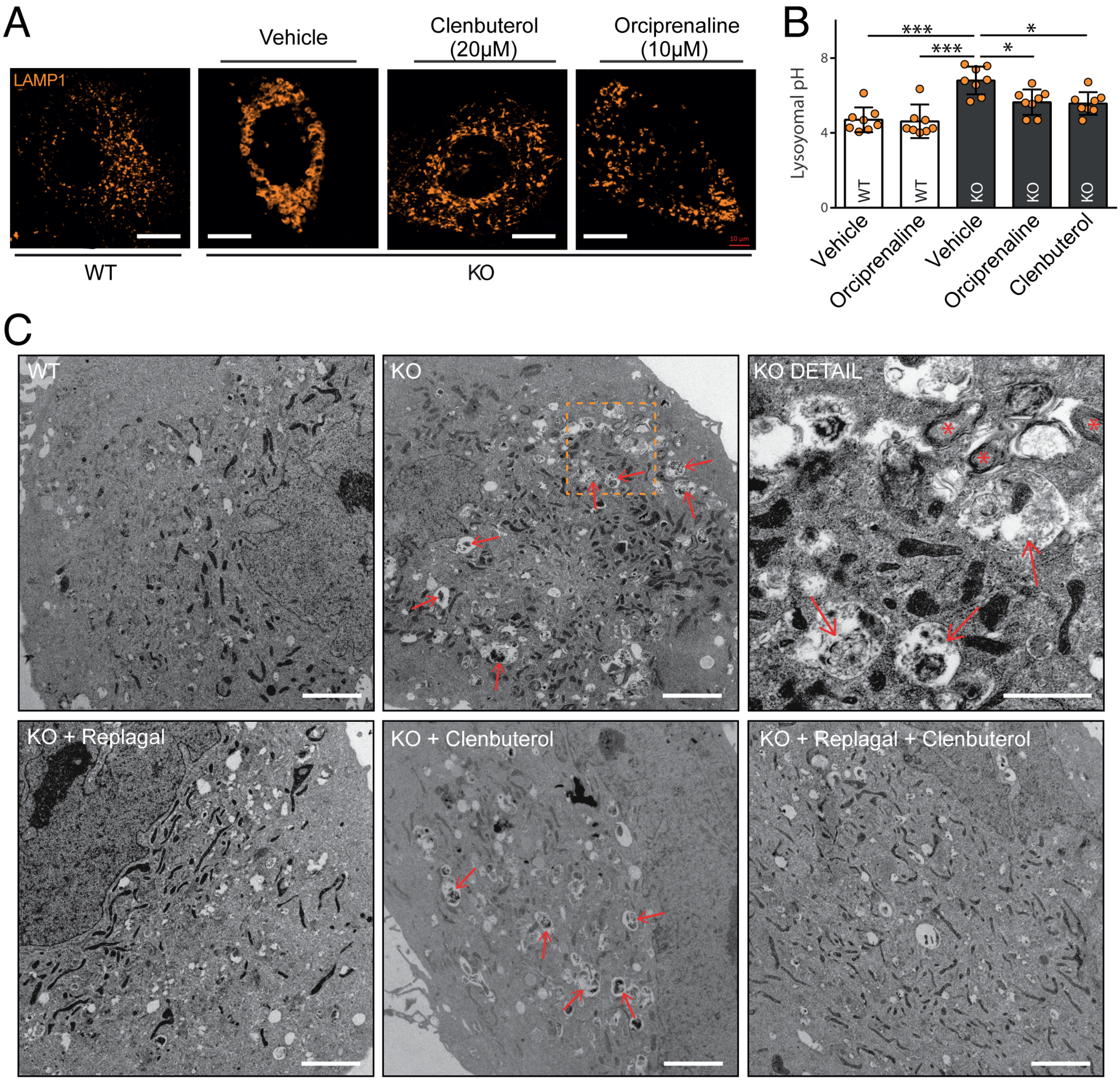

Figure S9
(A) LAMP-1 staining in WT (D1), KO-A, KO-A treated with 20µM Clenbuterol and KO-A treated with 10 µM Orciprenaline shows a partial restoration of subcellular lysosomal distribution in beta 2 agonist treated KO-A cells compared to untreated ones. Scalebars indicating 10µm. (B) The quantitative ratiometric LysoSensor yellow/blue DND-160 assay demonstrates the beneficial significant effect of β2 adrenergic receptor agonists on Lysosomal pH (N=8). (C) Representative TEM images showing the ultrastructural change in the different treatment groups. Wild type cells treated with PBS. GLA knock out cells treated with PBS demonstrating drastic accumulation of multilaminar vacuoles. GLA-KO cells treated with aGAL showing a restoration of the cellular ultrastructure. KO cells treated with 20µM Clenbuterol present a moderate decrease in the multilaminar vacuoles KO cells treated with recombinant aGAL and Clenbuterol showing a full restoration of the cellular ultrastructure. Cells in all groups were differentiated over 6 days and treated afterwards over 96 hours. Scalebars indicating 3µm and 1µm in detail.

Full unedited gel for Figure 1C GLA

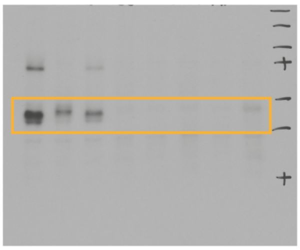

Full unedited gel for Figure 1C Tub

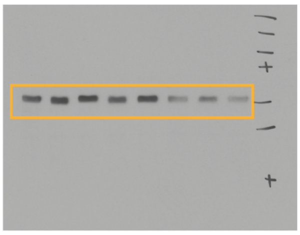

Full unedited gel for Figure 3B & S5 SncA

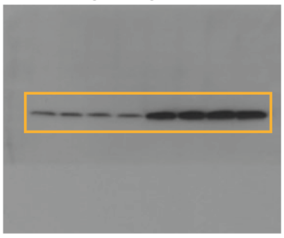

Full unedited gel for Figure 3B & S5 TubA

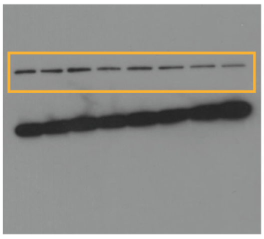

Full unedited gel for Figure 4A SncA

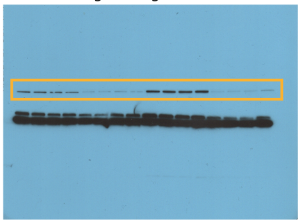

Full unedited gel for Figure 4A TubA

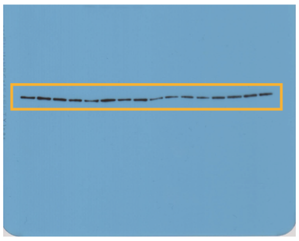

Full unedited gel for Figure 4C SncA

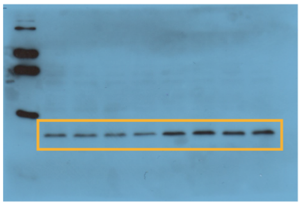

Full unedited gel for Figure 4C TubA

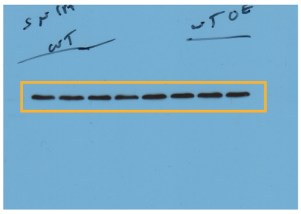

Full unedited gel for Figure 4G SncA

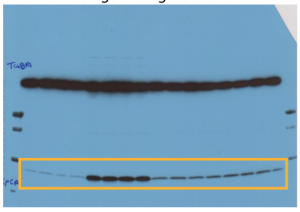

Full unedited gel for Figure 4G Actn4

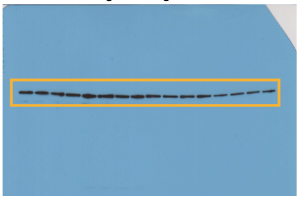

Full unedited gel for Figure 4H Lamp1

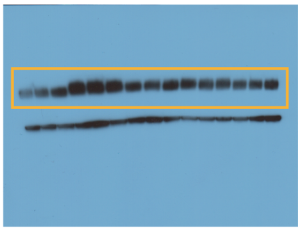

Full unedited gel for Figure 4H Actn4

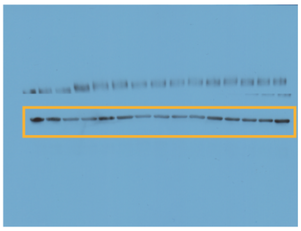

Full unedited gel for Figure S1B Gb3

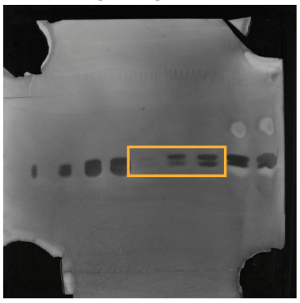

Full unedited gel for Figure S2A p62

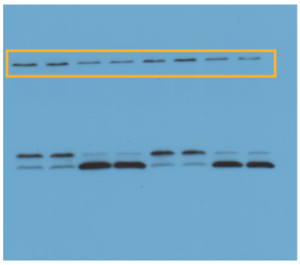

Full unedited gel for Figure S2A Lc3

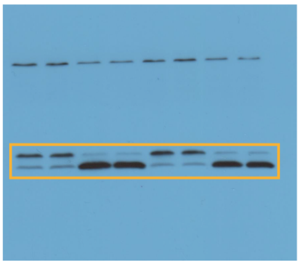

Full unedited gel for Figure S2A TubA

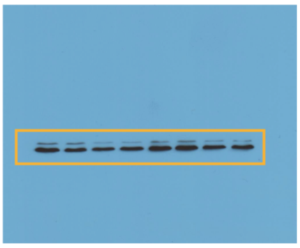

Full unedited gel for Figure S5 Ttyh3

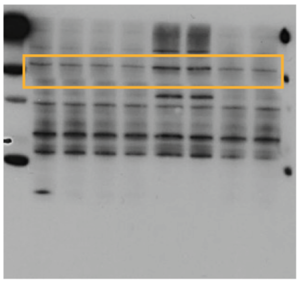

Full unedited gel for Figure S5 Scarb2

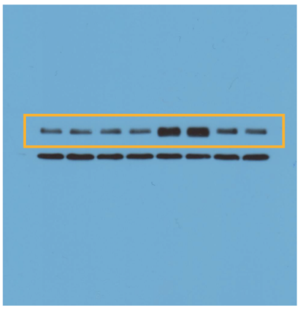

Full unedited gel for Figure S5 Plscr3

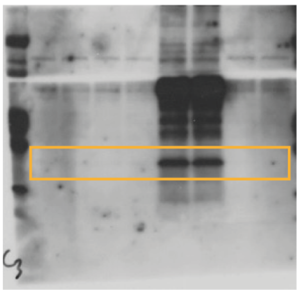

Full unedited gel for Figure S5 Mfge8

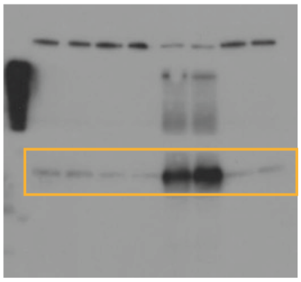

Full unedited gel for Figure S5 Gba

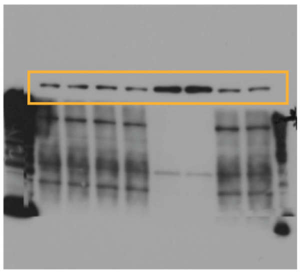

Full unedited gel for Figure S5 CD63

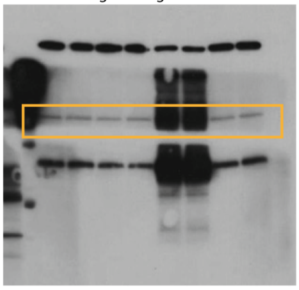

Full unedited gel for Figure S5 Itm2B

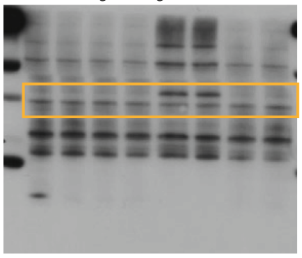

Full unedited gel for Figure S5 Dpp7

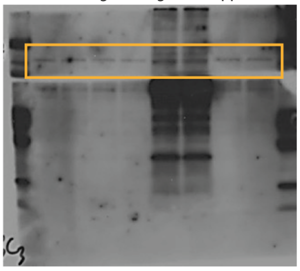

Full unedited gel for Figure S8A SncA

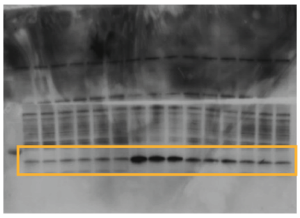

Full unedited gel for Figure S8A Actn4

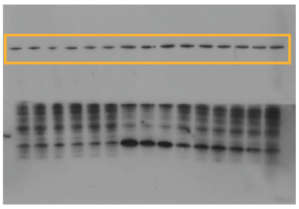

Supplement: Supplemental data [file jci-133-157782-s179.pdf]
